# Systemic therapy in children and adolescents with mental disorders: a systematic review and meta-analysis

**DOI:** 10.1186/s12888-024-05556-y

**Published:** 2024-02-14

**Authors:** David Henry Seidel, Martina Markes, Ulrich Grouven, Claudia-Martina Messow, Wiebke Sieben, Marco Knelangen, Rieke Oelkers-Ax, Sebastian Grümer, Heike Kölsch, Mandy Kromp, Markus von Pluto Prondzinski

**Affiliations:** 1 Institute for Quality and Efficiency in Health Care (Institut für Qualität und Wirtschaftlichkeit im Gesundheitswesen, IQWiG), Im Mediapark 8, Cologne, 50670 Germany; 2 Family Therapy Centre (Familientherapeutisches Zentrum gGmbH, FaTZ), Hermann-Walker-Straße 16, 69151 Neckargemünd, Germany

**Keywords:** Psychotherapy, Systemic therapy, Cognitive behavioural therapy, Mental disorders, Child, Adolescent, Benefit assessment, Systematic review

## Abstract

**Background:**

Systemic therapy (ST) is a psychotherapeutic intervention in complex human systems (both psychological and interpersonal). Cognitive behavioural therapy (CBT) is an established treatment for children and adolescents with mental disorders. As methodologically rigorous systematic reviews on ST in this population are lacking, we conducted a systematic review and meta-analysis to compare the benefit and harm of ST (and ST as an add-on to CBT) with CBT in children and adolescents with mental disorders.

**Methods:**

We searched MEDLINE, Embase, PsycINFO and other sources for randomised controlled trials in 14 mental disorder classes for the above comparisons in respect of effects on patient-relevant outcomes (search date: 7/2022). Where possible, meta-analyses were performed and results were graded into 3 different evidence categories: “proof”, “indication”, or “hint” (or none of these categories). PRISMA standards were followed.

**Results:**

Fifteen studies in 5 mental disorder classes with usable data were identified. 2079 patients (mean age: 10 to 19 years) were analysed. 12/15 studies and 29/30 outcomes showed a high risk of bias. In 2 classes, statistically significant and clinically relevant effects in favour of ST were found, supporting the conclusion of a hint of greater benefit of ST for mental and behavioural disorders due to psychoactive substance use and of ST as an add-on to CBT for obsessive-compulsive disorders. In 2 other classes (eating disorders; hyperkinetic disorders), there was no evidence of greater benefit or harm of ST. For affective disorders, a statistically significant effect to the disadvantage of ST was found for 1 outcome, supporting the conclusion of a hint of lesser benefit of ST.

**Conclusions:**

Our results show a hint of greater benefit of ST (or ST as an add-on to CBT) compared with CBT for 2 mental disorder classes in children and adolescents (mental and behavioural disorders due to psychoactive substance use, obsessive compulsive disorders). Given the importance of CBT as a control intervention, ST can therefore be considered a beneficial treatment option for children and adolescents with certain mental disorders. Limitations include an overall high risk of bias of studies and outcomes and a lack of data for several disorders.

**Supplementary Information:**

The online version contains supplementary material available at 10.1186/s12888-024-05556-y.

## Background

Systemic therapy (ST) or systems-oriented therapy can be defined as a psychotherapeutic intervention in complex human systems (both psychological and interpersonal) [1]. ST focuses on relationships and interactions, and include a contextual view of the problem with circular models of pathogenesis. ST therefore often involves working with patients and their families, as well as significant others such as teachers and friends, without being restricted to a particular setting [2].

Prior to the publication of 3 systematic reviews in 2013 and 2017 [2–4], there were no evidence syntheses on ST for a wide range of mental disorders in children and adolescents. The 2 reviews by von Sydow et al. and Retzlaff et al. [2, 3] compared ST with other or no psychotherapeutic interventions across a wide range of mental disorders and associated conditions (e.g. juvenile delinquency). Positive effects of ST were shown in individual studies for different disorders, but no meta-analyses were conducted to support these findings. Riedinger et al. [4] compared ST with other or no psychotherapeutic interventions for mental disorders and conducted meta-analyses showing positive effects of ST for some disorders. However, they did not analyse individual outcomes and did not compare specific interventions with each other. They concluded that “more research is needed before more general conclusions about the effects of ST can be drawn” [4].

In Germany, decisions on the reimbursement of health care services by the statutory health insurance (SHI) funds are made by the highest decision-making body in the health care system, the Federal Joint Committee (Gemeinsamer Bundesausschuss, G-BA) [5]. The G-BA regularly commissions the German health technology assessment (HTA) agency, the Institute for Quality and Efficiency in Health Care (Institut für Qualität und Wirtschaftlichkeit im Gesundheitswesen, IQWiG) [6], to provide the scientific basis for these decisions in the form of HTA reports.

Psychoanalysis, psychodynamic therapy, and cognitive behavioural therapy (CBT) have been available as SHI outpatient services for children, adolescents and adults for decades [7]; ST for adults followed in 2020 after an IQWiG HTA report [8].

In 2023, IQWiG published another HTA report to inform the (still pending) decision on whether outpatient ST should also be reimbursed for children and adolescents with mental disorders [9]. The full HTA report assessed the benefit and harm of ST compared with several control interventions. This article presents the comparison between ST and CBT, which was chosen as the control intervention for presentation because it is the most common form of psychotherapy offered to children and adolescents in Germany in the outpatient sector [10] and because previous research suggests that it is superior to placebo (e.g. [11–13]). CBT therefore sets a relatively high bar for demonstrating benefit, so comparisons that include CBT as a control intervention are particularly informative.

The aim of this systematic review was to compare the benefit and harm of ST with CBT (and of ST as an add-on to CBT with CBT alone) in children and adolescents with mental disorders.

## Methods

### General information

IQWiG’s general methodological approach is described in its methods paper [14].

This systematic review was part of the German-language HTA report mentioned above [9]. The (German-language) protocol [15] (not registered in a protocol database) was published on the IQWiG website before the actual HTA was conducted; the HTA report [9] is also published there. This protocol also applies to the present systematic review. The HTA report includes data on a wide range of mental disorders. This review only considers mental disorders for which data on ST versus CBT (or ST as an add-on to CBT versus CBT alone) are available. Only completed studies were used, so ethical approval and patient consent were not required. We adhered to the PRISMA statement [16, 17] throughout the manuscript. Our description of methods broadly follows that in previous journal articles on IQWiG reviews ([18, 19], Supplementary file 1: Additional file 1).

### Study eligibility

We included both published and previously unpublished randomised controlled trials (RCTs) on ST versus CBT (or ST as an add-on to CBT versus CBT alone) in children and adolescents with mental disorders and investigating at least one predefined patient-relevant outcome [9]. In this context, the term “patient-relevant” refers to “how a patient feels, functions or survives” [20] and includes the categories of mortality, morbidity, and health-related quality of life [14].

Eligible studies included patients with any mental disorder listed in one of the established diagnostic classification systems such as the International Statistical Classification of Diseases and Related Health Problems 10th Revision (ICD-10, [21]), the Diagnostic and Statistical Manual of Mental Disorders 5th Edition (DSM-5, [22]) or any of their previous versions. There was no lower age limit for patients; the upper age limit was 21 years.

The studies had to examine psychotherapeutic interventions classified in the literature as systemic therapy [23–27]. The inclusion of a specific intervention was decided on the basis of its name or description in the study publication or other sources of information. If it was not possible to assign the experimental intervention directly to a specific systemic approach mentioned in the literature, its description was evaluated independently by 2 reviewers (MvPP and MM or MvPP and SG) to decide on whether or not the intervention could be classified as systemic. They discussed their classification with an additional internal reviewer (e.g., DHS) or the external reviewer (ROA), a psychiatrist for children and adolescents and systemic therapist. Disagreements were resolved by consensus. The setting of an intervention (e.g., family or group setting) was irrelevant for classification. Systemic-integrative approaches (containing both systemic and not clearly systemic components) were also considered. To be included in the review, they either had to be described in the literature as a systemic or systemic-integrative approach or it had to be clear from the description that the approach was predominantly systemic.

The control intervention was CBT. If the control intervention included both CBT and non-CBT components, the former had to predominate.

Co-interventions had to be similar between groups. CBT could also be a co-intervention; in this case, ST was investigated as an add-on to CBT compared with CBT alone.

Two comparisons were therefore examined:

1. ST versus CBT
2. ST as an add-on to CBT versus CBT alone

The detailed inclusion criteria are listed in Table 1.

**Table 1:** Inclusion criteria^a^

I-1	Population: Children and/or adolescents < 21 years of age with a mental disorder diagnosis with ICD-10 [21] or DSM-5 [22] (or previous versions) or other criteria valid enough that a diagnosis of a mental disorder could be reliably assumed at baseline^b^.
I-2	Experimental intervention: Treatment with a psychotherapeutic intervention that can be attributed to systemic therapy. The experimental intervention was either purely systemic or systemic-integrative (contains both systemic and not clearly systemic components), with systemic elements predominating.
I-3	Comparison (control intervention): cognitive behavioural therapy. The control intervention included either only CBT or both CBT and non-CBT components (with CBT components predominating).
I-4	Outcome: Patient-relevant outcomes such as mortality, morbidity (symptoms, hospitalisation, and overall functioning, adverse events) or health-related quality of life were analysed.
I-5	Design: randomised controlled trial; there was no restriction regarding study duration.
I-6	Language: For non-German and non-English publications, an English-language title or abstract showing the relevance of the study had to be available.
I-7	Full publication available: In this context, a full publication also includes a study report in accordance with ICH E3 [28] or a study report meeting the criteria of the CONSORT statement [29] and allowing an assessment of the study, providing that the information on the study methods and results contained in these documents was not confidential.

*CONSORT* Consolidated Standards of Reporting Trials, *DSM-5* Diagnostic and Statistical Manual of Mental Disorders (5th edition), *I* inclusion criterion, *ICH* International Council for Harmonisation of Technical Requirements for Pharmaceuticals for Human Use, *ICD-10 *International Statistical Classification of Diseases and Related Health Problems (10th revision), *RCT* randomised controlled trial

^a^Translation of extract from [9]

^b^At baseline, a diagnosis of a mental disorder was required in at least 80% of the children and/or adolescents in each study

### Search strategy and study selection 

The following bibliographic databases and study registries were searched by an experienced information specialist: MEDLINE (1946 to 2022), Embase (1974 to 2022), PsycINFO (1806 to 2022), the Cochrane Central Register of Controlled Trials, ClinicalTrials.gov, and the International Clinical Trials Registry Platform (ICTRP) Search Portal. The peer-reviewed search strategy included a combination of subject headings and free texts, with terms such as “systemic therapy” and “family therapy” (see Supplementary file 1: Additional file 2 for the full search strategy). The last search was conducted on 12 July 2022.

In addition, the reference lists of relevant systematic reviews and HTA reports published between 2012 and 2021 were screened to identify further studies. Moreover, persons and parties who had submitted comments on the preliminary version of IQWiG’s HTA report [30] were asked to provide any additional relevant studies. Finally, documents submitted to the G-BA during the public hearing were also reviewed.

After removing duplicates, 2 reviewers independently screened the titles and abstracts of the retrieved citations to identify potentially eligible publications. The full texts of these articles were independently assessed by the same reviewers. Non-German or non-English full texts that appeared to be relevant based on the information in the abstract were translated. All documents retrieved from non-bibliographical sources were also checked for eligibility or relevant study information. Disagreements were resolved by consensus. Where necessary, authors were contacted to decide on the final inclusion or exclusion of studies.

### Data extraction

Data extraction and risk-of-bias assessment were always conducted by one reviewer and checked by another; disagreements were resolved by consensus. Details of the studies were extracted using standardised tables. We extracted information on:

1. Study characteristics, including the study design, length of follow-up, sample size, location, and period in which the study had been conducted
2. Characteristics of study participants, including inclusion and exclusion criteria, age, sex, diagnoses of mental disorders
3. Characteristics of the experimental and control interventions
4. Outcomes and type of outcome measures. Given the large number of potential outcomes to be assessed, some of the outcomes that were considered less important were excluded from the assessment before the respective results were examined.
5. Risk-of-bias items (see below).

Where necessary, authors were contacted to provide missing data or to clarify issues. For more details on the methods applied and the handling of missing data/dropouts, intention-to-treat (ITT) analyses, and scale assessments, please see previous IQWiG publications [9, 14].

### Risk‑of‑bias assessment

Using IQWiG’s methods paper [14] including Cochrane methods [31], the risk of bias at the study and outcome level was rated as high or low using the following items at the study level: generation of a randomisation sequence, allocation concealment, blinding of patients and health care professionals, reporting of all relevant outcomes irrespective of results, and other aspects. If the generation of a randomisation sequence or allocation concealment was judged to be inadequate, the other items at the study level were not assessed, because a high risk of bias at the study level was already apparent. A high risk of bias at the study level resulted in a high risk of bias at the outcome level. If the risk of bias at the study level was low, the following outcome-specific items were assessed: blinding of outcome assessors, use of the ITT principle, reporting of individual outcomes independent of results, and other aspects.

In a further step, we assessed the certainty of the study results and graded it as moderate or high, depending on the results of the risk-of-bias assessment.

### Grading of results

Using IQWiG’s methods [14], the results for each outcome were graded into 3 different evidence categories: “proof”, “indication”, or “hint” (or none of these categories) of greater benefit of the experimental intervention. In short, proof of greater benefit of the experimental intervention is inferred if a meta-analysis of at least 2 studies with a high certainty of results shows a statistically significant effect in favour of the experimental intervention. An indication of greater benefit is inferred if a single study with a high certainty of results shows a statistically significant effect in favour of the experimental intervention, or a meta-analysis of studies with a moderate certainty of results shows a statistically significant effect in favour of the experimental intervention. A hint of greater benefit is inferred if a single study with a moderate certainty of results shows a statistically significant effect in favour of the experimental intervention. No evidence (i.e., no proof, indication or hint) of greater benefit is inferred if there are no statistically significant differences between the experimental and control interventions, if the results are inconclusive, or if no suitable data are available. If studies with both a low and a high risk of bias are available for a given outcome, the studies with a low risk of bias are primarily used to derive evidence (i.e., proof, indication or hint) of greater benefit of ST. The above approach is also used to determine harm.

Based on each outcome assessment and using the same evidence categories, an assessment of the benefit and harm of ST was performed across outcomes, taking into account the clinical relevance of the outcomes and the strength of the evidence (in particular, effect sizes and consistency across effects for a given outcome).

### Data analysis

In the full HTA report [9], the studies included were grouped into classes that covered similar mental disorders. The grouping was based on a G-BA guideline that defines the criteria that a psychotherapeutic approach must fulfil in order to be offered as an SHI service [7]. This approach largely corresponds to the classification of mental disorders commonly used in established international diagnostic classification systems. The benefit of ST was assessed separately for the 14 different classes of mental disorders (plus the category “unspecified mental disorders”) included in the G-BA guideline.

If results for several different analysis points were available, the key analysis points were chosen (e.g., at baseline, mid-study, end-of-study, and follow-up). Odds ratios (OR) were calculated to compare dichotomous outcomes. Mean differences (MD) or Hedges’ g were calculated to compare continuous outcomes. In most cases, Hedges’ g was used to adjust for the different scales used to measure outcomes. A value of Hedges’ g of 0.2 (or − 0.2) was used as a clinical irrelevance threshold for continuous outcomes [32]. 95% confidence intervals (CIs) were reported for all effect estimates. To be assigned to one of the evidence categories “proof”, “indication” or “hint”, effects on continuous outcomes had to be not only statistically significant, but also considered clinically relevant.

Where possible and appropriate, data were pooled using meta-analyses. An overall effect was calculated using the Knapp and Hartung method with the Paule-Mandel heterogeneity estimator [33]. If 4 or fewer studies were available, a fixed-effect model was used to combine the study results. If relevant statistical heterogeneity [34] was present (Cochran’s Q test; *p* < 0.05), no overall effect estimate was calculated and, if possible, a 95% prediction interval [35] was calculated instead. The results of the meta-analysis were presented in forest plots. A *p*-value of < 0.05 was considered statistically significant.

Subgroup analyses were performed for age, sex, and type of systemic approach (systemic approaches only versus approaches combining systemic components with other components) if there were at least 10 patients with usable data and, in the case of binary data, at least 10 events per subgroup.

SAS software (version 9.4) was used for the data analysis.

## Results 

### Information retrieval and study selection 

**Fig. 1:**
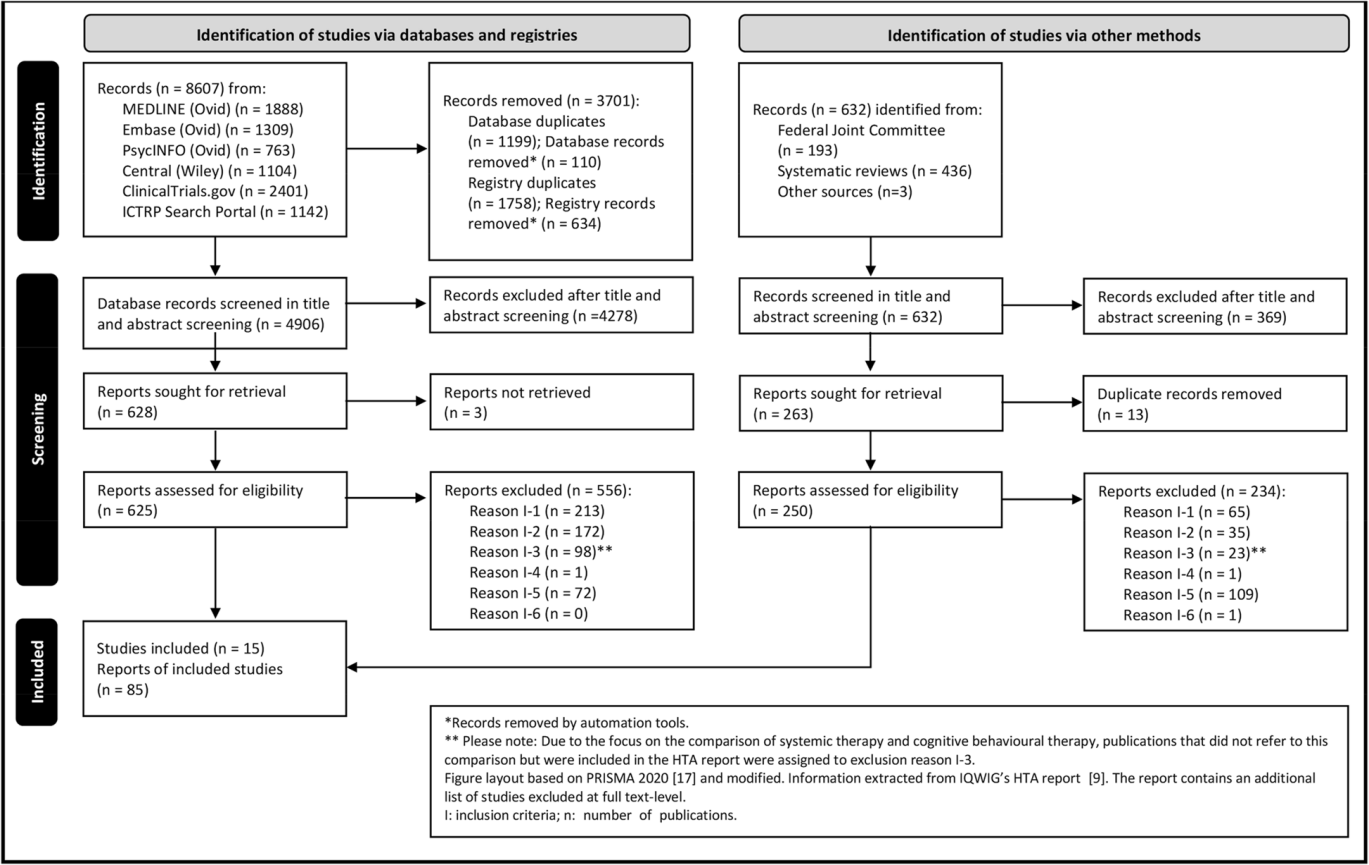
PRISMA flow chart and study selection

The selection of studies is shown in Fig. 1. A total of 15 RCTs (Table 2**)** with usable data were included ([36–123]; details of the study pool are provided in Supplementary file 1: Additional file 3).

**Table 2:** Study pool of included RCTs

No	Study	References	Comparison
Class of mental disorder I: Affective disorders			
1	Brent 1997	[40–53]	ST versus CBT
Class of mental disorder II: Anxiety disorders and obsessive-compulsive disorders			
2	Lebowitz 2020	[86]	ST versus CBT
3	Peris 2013	[105–108]	ST as an add-on to CBT versus CBT alone
4	Siqueland 2005	[116]	ST as an add-on to CBT versus CBT alone
Class of mental disorder III: Eating disorders			
5	Le Grange 2015	[87–91]	ST versus CBT
6	Nyman-Carlsson 2019	[102–104]	ST versus CBT
7	Schmidt 2007	[112–115]	ST versus CBT
Class of mental disorder IV: Hyperkinetic disorders			
8	Boyer 2015	[36–39]	ST versus CBT
Class of mental disorder V: Mental and behavioural disorders due to psychoactive substance use			
9	CYT	[54–60]	ST versus CBT
10	Dakof 2015	[61, 62]	ST versus CBT
11	INCANT	[63–85]	ST versus CBT
12	Liddle 2008	[92–99]	ST versus CBT
13	Liddle 2018	[100, 101]	ST versus CBT
14	Slesnick 2013	[117–121]	ST versus CBT
15	Waldron 2001	[122, 123]	ST versus CBT

*No *number

### General study characteristics 

The 15 studies with 2079 eligible randomised patients (range 11 to 600 per study) were conducted in the United States (*n* = 11) and Europe (*n* = 4) and published between 1997 and 2020. There were 972 patients in the experimental intervention group (range: 5 to 212 per study) and 1107 patients in the control group (range: 6 to 238 per study). About 55% of all patients were male, with 1 study (Nyman-Carlsson et al. [102–104]) including only women. The mean age of patients in the studies was between 10 and 19 years. More details on the study characteristics are provided in Supplementary file 1: Additional file 4.

### Risk-of-bias assessment and certainty of results

Twelve studies had a high risk of bias (Supplementary file 1: Additional file 5). Only Schmidt et al. [112–115], Boyer et al. [36–39], and INCANT [63–85] had a low risk of bias. At the outcome level, only 1 out of 30 outcomes had a low risk of bias and therefore a high certainty of results: “substance use detected by laboratory tests” (No. 27 in Supplementary file 1: Additional file 5) in INCANT [63–85]. All other 29 outcomes had a high risk of bias at the outcome level and therefore a moderate certainty of results.

### Main results

**Fig. 2:**
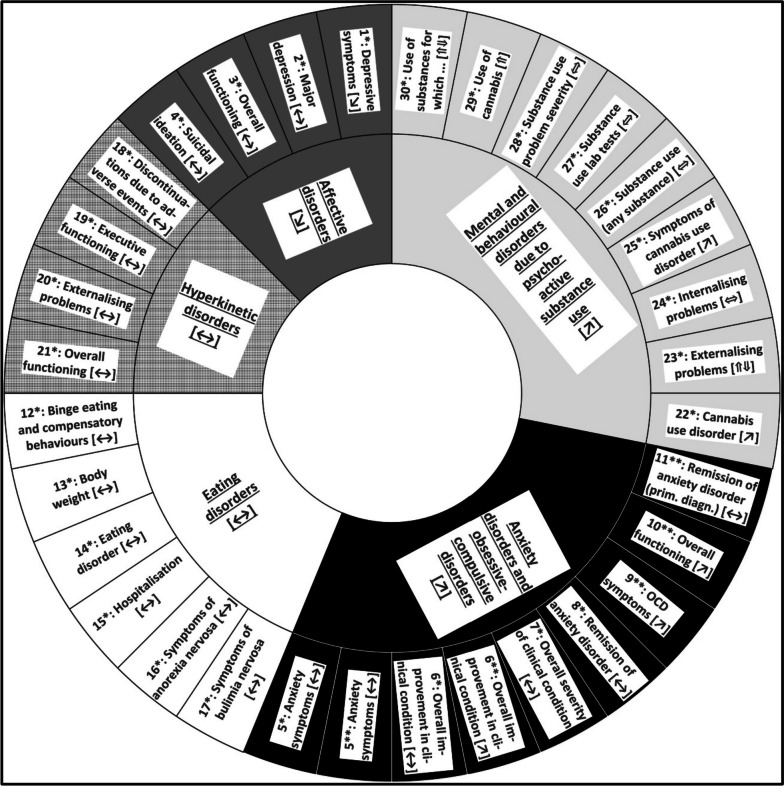
All conclusions on benefit for all classes of mental disorders (with usable data) and individual outcomes. [Legend: *: Comparison 1: ST versus CBT; **: Comparison 2: ST as an add-on to CBT versus CBT alone; [↘]: hint of lesser benefit of ST compared with CBT (based on a single study); [↗]: hint of greater benefit of ST (or ST as an add-on to CBT) compared with CBT (based on a single study); [ ↔]: no evidence (i.e. no proof, indication or hint) of greater benefit or harm of ST (or ST as an add-on to CBT) (based on a single study); [⇑]: indication of greater benefit of ST (supported by meta-analysis); [⇔]: no evidence of greater benefit or harm of ST (homogeneous results between studies); [⇑⇓]: no evidence of greater benefit or harm of ST (heterogeneous results between studies); OCD: obsessive-compulsive disorder; prim. diagn.: primary diagnosis; ST: systemic therapy; CBT: cognitive behavioural therapy. See Supplementary file 1: Additional file 6 for full definitions of outcomes

A total of 30 patient-relevant outcomes were identified in 5 classes of mental disorders with usable data (Fig. 2). Some outcomes either yielded heterogeneous results that did not allow the pooling of data or the identification of clear directions of effect, or significant results exceeded clinical irrelevance thresholds (± 0.2) and were therefore considered irrelevant (i.e. 95% CI covers − 0.2 or 0.2). No studies with usable data on the experimental and control intervention (ST vs. CBT; ST as an add-on to CBT vs. CBT) included could be identified for unspecified mental disorders or 9 further classes of mental disorders specified in the G-BA guideline [7], namely 1) conduct disorders, 2) pervasive developmental disorders, 3) somatoform disorders and dissociative disorders (conversion disorders), 4) reaction to severe stress, and adjustment disorders, 5) non-organic sleep disorders, 6) sexual dysfunction, 7) personality disorders and conduct disorders, 8) mental illnesses as a result of severe chronic diseases, and 9) schizophrenic and affective psychotic disorders. Table 3 shows the main results for the comparison of ST and CBT, i.e. those that support the conclusion that the data provide evidence (i.e. proof, indication or hint) of greater or lesser benefit of ST for a given outcome. Table 4 shows the main results for the comparison of ST as an add-on to CBT and CBT alone. More details are provided in the following sections and in Supplementary file 1: Additional file 6, including 18 forest plots.

**Table 3:** Statistically significant effects for outcomes for which the data provided evidence (proof, indication or hint) of greater or lesser benefit of ST compared with CBT (from [9])

Outcome		Operationalisation	Length of follow-up	Effect measure	Effect esti-mate	95% CI	*p*-value	Study	Direction of effect
**I: Affective** **disorders**									
No.1	Depressive symptoms	Beck Depression Inventory < 9	Week 12 – 16	OR	0.35	[0.13; 0.97]	0.045	Brent et al. [40–53]	▼
**V: Mental** **and** **behavioural** **disorders** **due** **to** **psychoactive** **substance** **use**									
No. 22	Cannabis use disorder	Categories: remission, abuse, dependence (ADI-Light for cannabis)	Month 12	OR	1.68	[1.15; 2.44]	0.007	INCANT [63–85]	▲
No. 25	Symptoms of cannabis use disorder	Number of symptoms of cannabis use disorder (ADI-Light for cannabis)	Month 0 – 12	Cohen’s d	1.27	[0.51; 2.03]	< 0.001	INCANT [63–85]	▲
			Month 12	MD	− 0.60	[− 0.99; − 0.21]	0.003	INCANT [63–85]	
No. 29	Use of cannabis	Days of cannabis use days in the past 90 days, adolescent self-report (TLFB)	Month 12	MD	− 27.14	[− 44.24; − 10.04]	0.002	Waldron et al. [122, 123]	▲
			Month 6	MD	7.90	[− 14.45; − 1.35]	0,018	INCANT [63–85]	▲
			Month 6 / 7	MD	− 8.22	[− 14.40; − 2.03]	0.009	**Meta-analysis:** INCANT [63–85] Waldron et al. [122, 123]	▲
			Month 12	MD	− 8.30	[− 14.83; − 1.77]	0.013	INCANT [63–85]	▲
		% of cannabis use days in the past 90 days, adolescent self-report (FORM 90D, TLFB)	Month 4	n/a	n/a	n/a	< 0.025	Waldron et al. [122, 123]	▲

▲ Effect in favour of ST ▼ Effect to the disadvantage of ST

*ADI-Light* Adolescent Diagnostic Interview-Light, *CI* confidence interval, *MD* mean difference, *n/a* not available or not specified in the study, *No.* number, *OR* odds ratio, *TLFB* timeline followback

**Table 4:** Statistically significant effects for outcomes for which the data provided evidence (proof, indication or hint) of greater or lesser benefit of ST as an add-on to CBT compared with CBT alone (from [9])

Outcome		Operationalisation	Length of follow-up	Effect measure	Effect esti-mate	95% CI	*p*-value	Study	Directionof effect
**II: Anxiety** **disorders and obsessive–compulsive** **disorders**									
No. 6	Overall improvement in clinical condition	CGI-I ≤ 2	Week 14	OR	3.15	[1.10; 8.99]	0.03	Peris et al. [105–108]	▲
No. 9	Obsessive-compulsive disorder symptoms	CY-BOCS ≤ 14	Week 14	OR	3.81	[1.29; 11.20]	0.01	Peris et al. [105–108]	▲
No. 10	Overall functioning	COIS-R	Week 14	Hedges’ g	− 0.75	[− 1.27; − 0.23]	0.004	Peris et al. [105–108]	▲

▲ Effect in favour of ST

*CI* confidence interval, *CGI-I* Clinical Global Impression – Improvement, *COIS-R* Child Obsessive Compulsive Impact Scale-Revised, *CY-BOCS* Children's Yale-Brown Obsessive Compulsive Scale, *MD* mean difference, *No.* number, *OR* odds ratio

### Comparison 1: ST versus CBT

For the comparison of ST and CBT, data were available for 5 classes of mental disorders:

### Class I: Affective disorders

One relevant study was identified (Brent et al. [40–53]); 2 of the 3 study arms (72 patients) compared ST with CBT in patients with major depression. The mean age of the patients was 16 years (range 13 to 18 years). The length of follow-up ranged from 12 weeks to 28 months.

Data were reported for 4 outcomes (Nos. 1 to 4 in Supplementary file 1: Additional file A6.1). For the dichotomous outcome “depressive symptoms” (Beck Depression Inventory [BDI] < 9), there was a statistically significant effect to the disadvantage of ST (OR = 0.35; 95% CI: [0.13; 0.97], *p* = 0.045; Table 3). No statistically significant effects were found for the other outcomes or operationalisations. However, most of the respective point estimates showed effects to the disadvantage of ST. A meta-analysis was not performed because only one study was available.

For depressive symptoms, the data provided a hint of lesser benefit of ST compared with CBT, whereas for the other outcomes there was no evidence (i.e. no proof, indication or hint) of greater or lesser benefit of ST; however, the point estimates largely indicated a disadvantage of ST.

Overall, the results support the conclusion that there is a hint of lesser benefit of ST compared with CBT for affective disorders.

### Class II: Anxiety disorders

One study including 124 patients was identified (Lebowitz et al. [86]). The study investigated ST in patients with an anxiety disorder and compared ST with CBT. The mean age of patients was 10 years (range 7 to 14 years). The length of follow-up was 12 weeks.

The study reported data on 4 outcomes (Nos. 5 to 8 in Supplementary file 1: Additional file A6.2). No statistically significant or clinically relevant effects were found. A meta-analysis was not performed because only one study was available.

The data provided no evidence (i.e. no proof, indication or hint) of greater or lesser benefit of ST for any of the 4 outcomes.

Overall, the results support the conclusion that there is no evidence of greater benefit or harm of ST for anxiety disorders.

### Class III: Eating disorders

Three relevant studies were identified comparing ST with CBT in patients with anorexia nervosa (Nyman-Carlsson et al. [102–104]), bulimia nervosa (2 out of 3 study arms in Le Grange et al. [87–91]), and bulimia nervosa and an eating disorder not otherwise specified (Schmidt et al. [112–115]). The sample sizes of the relevant populations ranged from 78 to 110 patients per study (total: 273 patients). The mean age of the patients was between 16 and 19 years (range across all 3 studies: 12 to 24 years). The length of follow-up ranged from 8 weeks to 36 months.

The 3 studies reported data on 6 outcomes (Nos. 12 to 17 in Supplementary file 1: Additional files A6.4, A6.5).

Results for hospitalisation were reported in 2 studies (Le Grange et al. [87–91] and Nyman-Carlsson et al. [102–104]). No pooled effect estimate was calculated due to relevant statistical heterogeneity. As only Le Grange et al. showed a statistically significant effect, it was not possible to conclude an effect on hospitalisation (see Section 3.1.4 in [14]).

Two studies (Le Grange et al. [87–91] and Schmidt et al. [112–115]) provided data on binge eating and compensatory behaviours, including 10 different operationalisations. The respective effects were either not statistically significant or statistically significant, but in opposite directions. Therefore, no clear conclusion about a benefit of ST could be drawn for this outcome.

No statistically significant effects were found for the other outcomes. No pooled effect estimates were calculated because of relevant statistical heterogeneity or because only results from 1 study were available for each outcome, operationalisation or analysis point.

The data provided no evidence (i.e. no proof, indication or hint) of greater or lesser benefit of ST for any of the 6 outcomes.

Overall, the results support the conclusion that there is no evidence of greater benefit or harm of ST for eating disorders.

### Class IV: Hyperkinetic disorders

One study including 159 patients investigated ST in patients with attention-deficit/hyperactivity disorder (Boyer et al. [36–39]). The mean age of the patients was 14 years (range 12 to 17 years). ST and CBT were provided as an add-on to motivational interviewing, psychoeducation and, in some cases, medication. The length of follow-up ranged from about 2 to 5 months.

The study reported data on 4 outcomes (Nos. 18 to 21 in Supplementary file 1: Additional file A6.6). No statistically significant or clinically relevant effects were found. A meta-analysis was not performed because only one study was available.

The data provided no evidence (i.e. no proof, indication or hint) of greater or lesser benefit of ST for any of the 4 outcomes.

Overall, the results support the conclusion that there is no evidence of greater benefit or harm of ST for hyperkinetic disorders.

### Class V: Mental and behavioural disorders due to psychoactive substance use

Seven studies investigated ST in patients with cannabis abuse or dependence (CYT [54–60], Dakof et al. [61, 62], INCANT [63–85], Liddle et al. 2008 [92–99]), cannabis use disorder (Liddle et al. 2018 [100, 101]), substance abuse (Waldron et al. [122, 123]) or abuse or dependence of alcohol or other substances (Slesnick et al. [117–121]). The sample sizes of the relevant populations ranged from 61 to 450 patients (total: 1378 patients). The mean age of the patients was 15 to 16 years (range across all included studies: 12 to 18 years). The length of follow-up ranged from 2 to 42 months. All studies compared ST with CBT. Three studies (CYT, Slesnick et al., and Waldron et al.) included additional study arms without CBT. In 6 studies, CBT was supplemented with measures to increase motivation, mostly motivational interviewing (CYT, Dakof et al., INCANT, Liddle et al. 2018, Slesnick et al., and Waldron et al.). In 1 study, CBT included components of dialectal behaviour therapy (Liddle et al. 2008). In 1 study (INCANT), all patients received CBT; almost half of these patients also received psychodynamic therapy. The control intervention in Slesnick et al. and one of the control interventions in CYT was the community reinforcement approach. This was a behavioural therapy intervention based on operant conditioning and included contingency management techniques, functional analysis, and skills training. Due to the high degree of overlap between the content of this approach and that of CBT, these control interventions were equated with CBT and included in the review.

The 7 studies reported data on 9 outcomes (Nos. 22 to 30 in Supplementary file 1: Additional files A6.7, A6.8). For 3 outcomes, there were statistically significant and clinically relevant effects in favour of ST (“cannabis use disorder”: OR = 1.68, 95% CI: [1.15; 2.44], *p* = 0.007; “symptoms of cannabis use disorder”: Cohen’s d = 1.27, 95% CI: [0.51; 2.03], *p* < 0.001; MD =  − 0.60, 95% CI [− 0.99; − 0.21] *p* = 0.003; and “use of cannabis”: MD =  − 27.14, 95% CI: [− 44.24; − 10.04], *p* = 0.002; MD =  − 8.22, 95% CI: [− 14.40; − 2.03], *p* = 0.009; MD =  − 8.30, 95% CI: [− 14.83; − 1.77], *p* = 0.013; *p* < 0.025; Table 3). For use of cannabis, we pooled data from 2 studies for 2 analysis points. For the different operationalisations of “use of substances for which criteria for a substance use disorder are not met”, effects were either not statistically significant or statistically significant, but in opposite directions. Therefore, no clear conclusion could be drawn for this outcome. For the other outcomes or operationalisations, the effects were either not statistically significant or not clinically relevant.

For externalising problems, internalising problems, substance use problem severity, and use of cannabis, the effects were pooled for several operationalisations. For the other outcomes, no pooled effect estimates were calculated because of relevant statistical heterogeneity or because results were available from only one study.

For cannabis use disorder, symptoms of cannabis use disorder, and use of cannabis, the data provided a hint of greater benefit of ST, whereas for the other outcomes there was no evidence (i.e. no proof, indication or hint) of greater or lesser benefit.

All effects in favour of ST were found in 2 studies (INCANT and Waldron et al.). In Waldron et al., the control intervention was explicitly described as individual CBT. In INCANT, all patients received CBT and only about half of these patients also received psychodynamic therapy. As all patients in Waldron et al. and the majority of patients in INCANT received CBT, the effects shown could be attributed with sufficient certainty to CBT. The results therefore support the conclusion of a hint of greater benefit of ST compared with CBT for mental and behavioural disorders due to psychoactive substance use.

### Comparison 2: ST as an add-on to CBT versus CBT alone

For the comparison of ST as an add-on to CBT and CBT alone, data were available only for anxiety disorders and obsessive-compulsive disorders (OCD):

### Class II: Anxiety disorders and obsessive-compulsive disorders

Two relevant studies including 73 patients (range: 11 to 62) were identified. They investigated ST in patients with an anxiety disorder (Siqueland et al. [116]) or an OCD (Peris et al. [105–108]). The mean age of patients was between 13 to 15 years (range across both studies 8 to 18 years). In the 2 studies, ST as an add-on to CBT was compared with CBT alone. The length of follow-up ranged from 14 weeks to 13 months.

The 2 studies reported data on 5 outcomes (Nos. 5 to 11 in Supplementary file 1: Additional file A6.3). There was a statistically significant effect in favour of ST as an add-on to CBT for 3 outcomes: “OCD symptoms” measured with the Children's Yale-Brown Obsessive Compulsive Scale (CY-BOCS; score ≤ 14): OR = 3.81, 95% CI [1.29; 11.20], *p* = 0.01; “overall functioning” measured with the Child Obsessive Compulsive Impact Scale-Revised (COIS-R): Hedges’ g =  − 0.75, 95% CI: [− 1.27; − 0.23], *p* = 0.004; and “overall improvement in clinical condition” measured with the Clinical Global Impression – Improvement (CGI-I; score ≤ 2): OR = 3.15, 95% CI: [1.10; 8.99], *p* = 0.03 (Table 4). While OCD symptoms and overall improvement in clinical condition were dichotomous outcomes, overall functioning was a continuous outcome, with the effect classified as clinically relevant because the upper limit of the 95% CI was below the irrelevance threshold of Hedges’ g − 0.2. No statistically significant or clinically relevant effects were found for the other outcomes or operationalisations. A meta-analysis was not performed because only one study was available for each outcome, operationalisation or analysis point.

For overall improvement in clinical condition, OCD symptoms and overall functioning (Nos. 6, 9 and 10 in Supplementary file 1: Additional file A6.3), the data provided a hint of greater benefit of ST as an add-on to CBT, whereas for the other outcomes there was no evidence (i.e. no proof, indication or hint) of greater or lesser benefit of ST as an add-on to CBT.

Overall, for patients with OCD, the results support the conclusion that there is a hint of greater benefit of ST as an add-on to CBT compared with CBT alone. As the effects in favour of ST were all shown in a study investigating OCD, the conclusion about the benefit of ST is limited to OCD.

## Discussion

### Summary of results

For 1 of the 14 mental disorder classes investigated (mental and behavioural disorders due to psychoactive substance use), an assessment across all outcomes showed a hint of greater benefit of ST compared with CBT. For eating disorders and hyperkinetic disorders, there was no evidence (i.e. no proof, indication or hint) of greater benefit or harm of ST. For affective disorders, there was a hint of lesser benefit of ST. Despite this null finding, the overall result for ST is considered positive because of the above-mentioned importance of CBT as a control intervention and because positive effects of ST were shown in the mental disorder class with the largest sample size, mental and behavioural disorders due to psychoactive substance use (1378 patients). In this class, there was a hint of greater benefit of ST compared with CBT across all outcomes. The conclusion has a reasonable degree of certainty because it is based on the results of 8 studies. The fact that there was no evidence of greater benefit or harm of ST in 2 classes of mental disorders (eating disorders and hyperkinetic disorders) should not be equated with no benefit at all compared with other treatments, especially as the present review only compared ST with CBT.

For 1 of the classes (OCD) an assessment across all outcomes showed a hint of greater benefit of ST as an add-on to CBT compared with CBT alone. However, with regard to the benefit of ST, this result is not as informative as the results comparing ST with CBT. In addition, the conclusion about the benefit of ST is limited to OCD, as evidence of a benefit of ST as an add-on to CBT was only found in the study on patients with OCD; no such evidence was found in the study on patients with an anxiety disorder.

To some extent the hint of less benefit of ST in affective disorders poses a problem for the otherwise positive body of evidence, as affective disorders are a particularly important class in terms of prevalence. However, this finding was based on the results of only a small study with 72 patients in the relevant population, so the conclusion on affective disorders should be viewed with caution.

### Comparison with previous research 

Due to methodological differences between our systematic review and previous systematic reviews [2–4], the comparability of results is limited.

Notably, none of the reviews included separate comparisons of ST with CBT. In addition, no meta-analyses were performed in von Sydow et al. [3] and Retzlaff et al. [2]. Riedinger et al. [4] performed meta-analyses, but unlike in our review, results were presented only for mental disorder classes, not for specific outcomes or specific comparisons of interventions. Moreover, the study pools of the 3 reviews and our review differed considerably due to the more stringent inclusion criteria in our review (confirmed diagnosis of a mental disorder required) and the more recent (5 to 9 years) literature search. We therefore believe that our review adds robust new evidence to the literature on ST.

### Strengths and limitations

As previous research has shown that CBT is superior to placebo [11–13], the comparison between ST and CBT is particularly informative with regard to the benefit of ST. Accordingly, comparing ST with CBT helps to reduce the risk of overestimating the effects of ST.

With the exception of mental and behavioural disorders due to psychoactive substance use (1378 patients) the sample sizes for the other mental disorder classes were relatively small (range: 72 to 273 patients) and meta-analyses were not feasible for 3 out of the 5 classes with usable data. We tried to reduce the variation in follow-up periods between the included studies by combining similar follow-up periods into one period in the meta-analyses. Although this made it impossible to draw conclusions about some individual follow-up periods, it increased the overall robustness and validity of the results. In addition, most of the studies had a high risk of bias at the study level (largely due to inadequate or unclear randomisation or allocation concealment), which also led to a high risk of bias at the outcome level. In addition, patients and therapists were not blinded, as blinding is hardly possible with this type of intervention, which also contributes to a high risk of bias. Due to the small number of studies in the analyses, no formal tests for publication bias were performed, which should be taken into account when interpreting the data. Positive conclusions about the benefit of ST could only be drawn for 2 mental disorder classes in this review; it is unclear whether these conclusions apply to other classes due to a lack of appropriate data. In addition, our conclusions apply only to the comparison of ST and CBT, and the comparison of ST as an add-on to CBT and CBT alone, not to ST in general. In fact, the full HTA report [9] compared ST with a range of non-CBT control interventions (or no intervention). For both eating disorders and hyperkinetic disorders, ST showed a benefit compared with non-CBT control interventions. Finally, in all studies the reporting of adverse events was incomplete; therefore, we were not able to comprehensively weigh the benefits and harms of ST. As most studies on psychotherapeutic interventions still lack a standardised approach for adverse event recording [124], this problem should be addressed in future research.

No data on the comparisons included were available for conduct disorders, pervasive developmental disorders and unspecified mental disorders (for which data on the non-CBT control interventions were available in the full HTA report [9]). In addition, no studies were found for the remaining classes of mental disorders specified in the G-BA guideline [7], namely, 1) somatoform disorders and dissociative disorders (conversion disorders), 2) reaction to severe stress, and adjustment disorders, 3) non-organic sleep disorders, 4) sexual dysfunction, 5) personality disorders and conduct disorders, 6) mental illnesses as a result of severe chronic diseases, and 7) schizophrenic and affective psychotic disorders.

## Conclusions

Our systematic review of ST for the treatment of children and adolescents with mental disorders shows a hint of greater benefit of ST (or ST as an add-on to CBT) compared with CBT for 2 classes of mental disorders: mental and behavioural disorders due to psychoactive substance use (ST) as well as OCD (ST + CBT), although the finding of greater benefit is less conclusive for OCD. Given the importance of CBT as a control intervention, ST may therefore be considered a beneficial treatment option for children and adolescents with certain mental disorders. Limitations of our review include an overall high risk of bias in the studies and outcomes analysed and a lack of data for several mental disorders. Results from high-quality RCTs are needed to confirm and extend our conclusions.

### Supplementary Information

**Supplementary file 1**: **Additional file 1.** PRISMA 2020 Checklist. **Additional file 2.** Search strategies. **Additional file 3.** Study pool of included RCTs. **Additional file 4.** Characteristics of included RCTs. **Additional file 5.** Risk-of-bias assessment. **Additional file 6.** All results.

## Data Availability

All data used in this article are available in the full German-language report published on the IQWiG website [9]: https://www.iqwig.de/en/projects/n21-03.html.

## Abbreviations

BDI: Beck Depression Inventory
CBT: Cognitive behavioural therapy
CGI-I: Clinical Global Impression – Improvement
COIS-R: Child Obsessive Compulsive Impact Scale-Revised
CY-BOCS: Children's Yale-Brown Obsessive-Compulsive Scale
DSM-5: Diagnostic and Statistical Manual of Mental Disorders 5th Edition
HTA: Health technology assessment
ICD-10: International Statistical Classification of Diseases and Related Health Problems 10th Revision
ICTRP: International Clinical Trials Registry Platform
IQWiG: Institute for Quality and Efficiency in Health Care
ITT: Intention to treat
G-BA: Federal Joint Committee (Gemeinsamer Bundesausschuss)
MD: Mean difference
No(s). / Nos.: Number(s)
OCD: Obsessive-compulsive disorder
OR: Odds ratio
PRISMA: Preferred Reporting Items for Systematic reviews and Meta-Analyses
RCT: Randomised controlled trial
RD: Risk difference
SHI: Statutory health insurance
ST: Systemic therapy
