# Supplementary material for: Systemic therapy in children and adolescents with mental disorders: a systematic review and meta-analysis
Source: BMC Psychiatry. 2024 Feb 14;24:125. doi: 10.1186/s12888-024-05556-y (PMC10868021; doi:10.1186/s12888-024-05556-y)
Supplement: Supplementary file 1 — Additional file 1. PRISMA 2020 Checklist. Additional file 2. Search strategies. Additional file 3. Study pool of included RCTs. Additional file 4. Characteristics of included RCTs. Additional file 5. Risk-of-bias assessment. Additional file 6. All results. [file 12888_2024_5556_MOESM1_ESM.pdf]

Systemic therapy in children and adolescents with mental disorders: a systematic review and meta-analysis

David Henry Seidel[1,*], Martina Markes[1], Ulrich Grouven[1], Claudia-Martina Messow[1], Wiebke Sieben[1], Marco Knelangen[1], Rieke Oelkers-Ax[2], Sebastian Grümer[1], Heike Kölsch[1], Mandy Kromp[1], Markus von Pluto Prondzinski[1]

[1] Institute for Quality and Efficiency in Health Care (Institut für Qualität und Wirtschaftlichkeit im Gesundheitswesen, IQWiG), Im Mediapark 8, 50670, Cologne, Germany

[2] Family Therapy Centre (Familientherapeutisches Zentrum gGmbH, FaTZ), Hermann-Walker-Straße 16, 69151 Neckargemünd, Germany

*Correspondence: david.seidel@iqwig.de

Table of content

Page

List of tables **3**
List of figures **4**
A1 Additional file 1 – PRISMA 2020 Checklist **6**
A2 Additional file 2 – Search strategies **10**
A2.1 Bibliographical databases **10**
Search for systematic reviews 10
1. MEDLINE via Ovid 10
2. The Cochrane Library 11
Search for primary studies 12
1. MEDLINE via Ovid 12
2. Embase via Ovid 14
3. PsycINFO via Ovid 15
4. The Cochrane Library via Wiley 16
A2.2 Study register **18**
1. ClinicalTrials.gov 18
2. International Clinical Trials Registry Platform Search Portal 18
A3 Additional file 3 – Study pool of included RTCs **19**
A4 Additional file 4 – Characteristics on included RCTs **20**
A5 Additional file 5 – Risk of bias assessment **30**
A6 Additional file 6 – All results **32**
A6.1 Tabular view – Class of mental disorders I: Affective disorders **32**
A6.2 Tabular view – Class of mental disorders II: Anxiety disorders **34**
A6.3 Tabular view – Class of mental disorders II: Anxiety disorders and obsessive-compulsive disorders **36**
A6.4 Tabular view – Class of mental disorders III: Eating disorders **39**
A6.5 Forest plots – Class of mental disorders III: Eating disorders **44**
A6.6 Tabular view – Class of mental disorders IV: Hyperkinetic disorders **45**
A6.7 Tabular view – Class of mental disorders V: Mental and behavioural disorders due to psychoactive substance use **48**
A6.8 Forest plots – Class of mental disorders V: Mental and behavioural disorders due to psychoactive substance use **58**
References **74**

List of tables

Page

Table 1: PRISMA 2020 Checklist 6

Table 2: Study pool of included RTCs (extended) 19

Table 3: Characteristics on included RCTs 20

Table 4: Risk of bias assessment – study level 30

Table 5: Risk of bias assessment – outcome level 31

Table 6: Affective disorders – results for all patient-relevant outcomes with assessable data 32

Table 7: Anxiety disorders – results for all patient-relevant outcomes with assessable data (comparison 1: ST versus CBT) 34

Table 8: Anxiety disorders and obsessive-compulsive disorders – results for all patient-relevant outcomes with assessable data (comparison 2: ST as an add-on to CBT versus CBT alone) 36

Table 9: Eating disorders – results for all patient-relevant outcomes with assessable data .. 39

Table 10: Hyperkinetic disorders – results for all patient-relevant outcomes with assessable data 45

Table 11: Mental and behavioural disorders due to psychoactive substance use – results for all patient-relevant outcomes with assessable data 48

List of figures

Page

Figure 1: Forest Plot for binge eating and compensatory behaviours after 6 months; Systemic Therapy (ST) vs. Cognitive Behavioural Therapy (CBT); Eating disorders 44

Figure 2: Forest Plot for externalising problems (YSR) after 4 or 6 months; Systemic Therapy (ST) vs. Cognitive Behavioural Therapy (CBT); Mental and behavioural disorders due to psychoactive substance use 58

Figure 3: Forest Plot for externalising problems (CBCL) after 6 months; Systemic Therapy (ST) vs. Cognitive Behavioural Therapy (CBT); Mental and behavioural disorders due to psychoactive substance use 59

Figure 4: Forest Plot for externalising problems (YSR) after 12 months; Systemic Therapy (ST) vs. Cognitive Behavioural Therapy (CBT); Mental and behavioural disorders due to psychoactive substance use 60

Figure 5: Forest Plot for externalising problems (CBCL) after 12 months; Systemic Therapy (ST) vs. Cognitive Behavioural Therapy (CBT); Mental and behavioural disorders due to psychoactive substance use 61

Figure 6: Forest Plot for externalising problems (YSR) after 18 months; Systemic Therapy (ST) vs. Cognitive Behavioural Therapy (CBT); Mental and behavioural disorders due to psychoactive substance use 62

Figure 7: Forest Plot for externalising problems (YSR) after 24 months; Systemic Therapy (ST) vs. Cognitive Behavioural Therapy (CBT); Mental and behavioural disorders due to psychoactive substance use 63

Figure 8: Forest Plot for internalising problems (YSR) after 4 or 6 months; Systemic Therapy (ST) vs. Cognitive Behavioural Therapy (CBT); Mental and behavioural disorders due to psychoactive substance use 64

Figure 9: Forest Plot for internalising problems (CBCL) after 6 months; Systemic Therapy (ST) vs. Cognitive Behavioural Therapy (CBT); Mental and behavioural disorders due to psychoactive substance use 65

Figure 10: Forest Plot for internalising problems (YSR) after 12 months; Systemic Therapy (ST) vs. Cognitive Behavioural Therapy (CBT); Mental and behavioural disorders due to psychoactive substance use 66

Figure 11: Forest Plot for internalising problems (CBCL) after 12 months; Systemic Therapy (ST) vs. Cognitive Behavioural Therapy (CBT); Mental and behavioural disorders due to psychoactive substance use 67

Figure 12: Forest Plot for internalising problems (YSR) after 18 months; Systemic Therapy (ST) vs. Cognitive Behavioural Therapy (CBT); Mental and behavioural disorders due to psychoactive substance use 68

Figure 13: Forest Plot for substance use problem severity after 4 or 6 months; Systemic Therapy (ST) vs. Cognitive Behavioural Therapy (CBT); Mental and behavioural disorders due to psychoactive substance use; missing standard deviation (SD) in INCANT (Switzerland) replaced by median of other studies 69

Figure 14: Forest Plot for substance use problem severity after 9 months; Systemic Therapy (ST) vs. Cognitive Behavioural Therapy (CBT); Mental and behavioural disorders due to psychoactive substance use; missing standard deviation (SD) in INCANT (Switzerland) replaced by median of other studies 70

Figure 15: Forest Plot for substance use problem severity after 12 months; Systemic Therapy (ST) vs. Cognitive Behavioural Therapy (CBT); Mental and behavioural disorders due to psychoactive substance use; missing standard deviation (SD) in INCANT (Switzerland) replaced by median of other studies 71

Figure 16: Forest Plot for substance use problem severity after 18 months; Systemic Therapy (ST) vs. Cognitive Behavioural Therapy (CBT); Mental and behavioural disorders due to psychoactive substance use 72

Figure 17: Forest Plot for use of cannabis after 3 or 4 months; Systemic Therapy (ST) vs. Cognitive Behavioural Therapy (CBT); Mental and behavioural disorders due to psychoactive substance use; missing patient numbers in Waldron 2001 replaced with randomised patients 72

Figure 18: Forest Plot for use of cannabis after 6 or 7 months; Systemic Therapy (ST) vs. Cognitive Behavioural Therapy (CBT); Mental and behavioural disorders due to psychoactive substance use 73

A1 Additional file 1 – PRISMA 2020 Checklist

retrieved on 09 February 2023 on https://prisma-statement.org/prismastatement/Checklist.aspx?AspxAutoDetectCookieSupport=1

Adopted from Page et al. 2021 (1) and completed

Table 1: PRISMA 2020 Checklist

Section and Topic	Item #	Checklist item	Location where item is reported
**TITLE**			
Title	1	Identify the report as a systematic review.	Manuscript: Title
**ABSTRACT**			
Abstract	2	See the PRISMA 2020 for Abstracts checklist.	Manuscript: General information
**INTRODUCTION**			
Rationale	3	Describe the rationale for the review in the context of existing knowledge.	Manuscript: Background
Objectives	4	Provide an explicit statement of the objective(s) or question(s) the review addresses.	Manuscript: Background
**METHODS**			
Eligibility criteria	5	Specify the inclusion and exclusion criteria for the review and how studies were grouped for the syntheses.	Manuscript: Study eligibility, Table 1
Information sources	6	Specify all databases, registers, websites, organisations, reference lists and other sources searched or consulted to identify studies. Specify the date when each source was last searched or consulted.	Manuscript: Search strategy and study selection
Search strategy	7	Present the full search strategies for all databases, registers and websites, including any filters and limits used.	A2 Additional file 2: Search strategies
Selection process	8	Specify the methods used to decide whether a study met the inclusion criteria of the review, including how many reviewers screened each record and each report retrieved, whether they worked independently, and if applicable, details of automation tools used in the process.	Manuscript: Search strategy and study selection, Fig. 1, Authors' contributions
Data collection process	9	Specify the methods used to collect data from reports, including how many reviewers collected data from each report, whether they worked independently, any processes for obtaining or confirming data from study investigators, and if applicable, details of automation tools used in the process.	Manuscript: Data extraction, Authors' contributions

Table 1: PRISMA 2020 Checklist

Section and Topic	Item #	Checklist item	Location where item is reported
Data items	10a	List and define all outcomes for which data were sought. Specify whether all results that were compatible with each outcome domain in each study were sought (e.g. for all measures, time points, analyses), and if not, the methods used to decide which results to collect.	Manuscript: Data extraction
	10b	List and define all other variables for which data were sought (e.g. participant and intervention characteristics, funding sources). Describe any assumptions made about any missing or unclear information.	Manuscript: Search strategy and study selection, Data extraction
Study risk of bias assessment	11	Specify the methods used to assess risk of bias in the included studies, including details of the tool(s) used, how many reviewers assessed each study and whether they worked independently, and if applicable, details of automation tools used in the process.	Manuscript: Risk-of-bias assessment, Authors' contributions
Effect measures	12	Specify for each outcome the effect measure(s) (e.g. risk ratio, mean difference) used in the synthesis or presentation of results.	Manuscript: Grading of results, Data analysis
Synthesis methods	13a	Describe the processes used to decide which studies were eligible for each synthesis (e.g. tabulating the study intervention characteristics and comparing against the planned groups for each synthesis (item #5)).	Manuscript: Study eligibility, Data analysis
	13b	Describe any methods required to prepare the data for presentation or synthesis, such as handling of missing summary statistics, or data conversions.	Manuscript: Grading of results, Data analysis
	13c	Describe any methods used to tabulate or visually display results of individual studies and syntheses.	Manuscript: Grading of results, Data analysis
	13d	Describe any methods used to synthesize results and provide a rationale for the choice(s). If meta-analysis was performed, describe the model(s), method(s) to identify the presence and extent of statistical heterogeneity, and software package(s) used.	Manuscript: Grading of results, Data analysis
	13e	Describe any methods used to explore possible causes of heterogeneity among study results (e.g. subgroup analysis, meta-regression).	Manuscript: Data analysis
	13f	Describe any sensitivity analyses conducted to assess robustness of the synthesized results.	Not required, as no sensitivity analysis is presented.
Reporting bias assessment	14	Describe any methods used to assess risk of bias due to missing results in a synthesis (arising from reporting biases).	Manuscript: Risk-of-bias assessment
Certainty assessment	15	Describe any methods used to assess certainty (or confidence) in the body of evidence for an outcome.	Manuscript: Risk-of-bias assessment, Grading of results

Table 1: PRISMA 2020 Checklist

Section and Topic	Item #	Checklist item	Location where item is reported
**RESULTS**			
Study selection	16a	Describe the results of the search and selection process, from the number of records identified in the search to the number of studies included in the review, ideally using a flow diagram.	Manuscript: Information retrieval and study selection; Fig. 1, Table 2
	16b	Cite studies that might appear to meet the inclusion criteria, but which were excluded, and explain why they were excluded.	Not relevant, as already published in the HTA Report (2).
Study characteristics	17	Cite each included study and present its characteristics.	Manuscript: Information retrieval and study selection, Table 2, A3, A4 Additional file 3, 4
Risk of bias in studies	18	Present assessments of risk of bias for each included study.	Manuscript: Risk-of-bias assessment and certainty of results; A5 Additional file 5
Results of individual studies	19	For all outcomes, present, for each study: (a) summary statistics for each group (where appropriate) and (b) an effect estimate and its precision (e.g. confidence/credible interval), ideally using structured tables or plots.	Manuscript: Main results, Fig. 2, Table 3, Table 4, A6 Additional file 6 – All results
Results of syntheses	20a	For each synthesis, briefly summarise the characteristics and risk of bias among contributing studies.	Manuscript: Risk-of-bias assessment and certainty of results; A5 Additional file 5
	20b	Present results of all statistical syntheses conducted. If meta-analysis was done, present for each the summary estimate and its precision (e.g. confidence/credible interval) and measures of statistical heterogeneity. If comparing groups, describe the direction of the effect.	Manuscript: Main results, Fig. 2, Table 3, Table 4, A6 Additional file 6 – All results
	20c	Present results of all investigations of possible causes of heterogeneity among study results.	Manuscript: Main results, Fig. 2, Table 3, Table 4, A6 Additional file 6 – All results
	20d	Present results of all sensitivity analyses conducted to assess the robustness of the synthesized results.	Not required, as no sensitivity analysis is presented.
Reporting biases	21	Present assessments of risk of bias due to missing results (arising from reporting biases) for each synthesis assessed.	Manuscript: Risk-of-bias assessment and certainty of results; A5 Additional file 5
Certainty of evidence	22	Present assessments of certainty (or confidence) in the body of evidence for each outcome assessed.	Manuscript: Main results, Fig. 2, Table 3, Table 4, A6 Additional file 6 – All results

Table 1: PRISMA 2020 Checklist

Section and Topic	Item #	Checklist item	Location where item is reported
**DISCUSSION**			
Discussion	23a	Provide a general interpretation of the results in the context of other evidence.	Manuscript: Summary of results; Comparison with previous research
	23b	Discuss any limitations of the evidence included in the review.	Manuscript: Strengths and limitations
	23c	Discuss any limitations of the review processes used.	Manuscript: Strengths and limitations
	23d	Discuss implications of the results for practice, policy, and future research.	Manuscript: Conclusions
**OTHER INFORMATION**			
Registration and protocol	24a	Provide registration information for the review, including register name and registration number, or state that the review was not registered.	Manuscript: General information
	24b	Indicate where the review protocol can be accessed, or state that a protocol was not prepared.	Manuscript: General information
	24c	Describe and explain any amendments to information provided at registration or in the protocol.	Manuscript: General information
Support	25	Describe sources of financial or non-financial support for the review, and the role of the funders or sponsors in the review.	Manuscript: Funding
Competing interests	26	Declare any competing interests of review authors.	Manuscript: Competing interests
Availability of data, code and other materials	27	Report which of the following are publicly available and where they can be found: template data collection forms; data extracted from included studies; data used for all analyses; analytic code; any other materials used in the review.	Manuscript: Availability of data and materials

A2 Additional file 2 – Search strategies

A2.1 Bibliographical databases

Search for systematic reviews

1. MEDLINE via Ovid

- Ovid MEDLINE(R) ALL 1946 to November 27, 2019

Adoption of the following search filters:

- Systematic reviews: Wong (3) – High specificity strategy

#	Searches
1	exp psychotherapy/
2	Stress, Psychological/th
3	psychotherap*.ti,ab.
4	or/1-3
5	(family adj3 (psychotherap* or therap* or intervention* or treatment*)).ti,ab.
6	(couple* adj1 (therap* or treatment*)).ti,ab.
7	((group or systemic* or strategic* or paradoxical* or conjoint* or marital* or multisystemic*) adj1 therap*).ti,ab.
8	(solution-focused* adj3 therap*).ti,ab.
9	or/5-8
10	and/4,9
11	cochrane database of systematic reviews.jn.
12	(search or MEDLINE or systematic review).tw.
13	meta analysis.pt.
14	or/11-13
15	14 not (exp animals/ not humans.sh.)
16	and/10,15
17	16 and (english or german).lg.
18	..l/ 17 yr=2012-Current

- Ovid MEDLINE(R) ALL 1946 to June 30, 2021

Adoption of the following search filters:

- Systematic reviews: Wong (3) – High specificity strategy

#	Searches
1	exp psychotherapy/
2	psychotherap*.ti,ab.
3	or/1-2
4	(family adj3 (psychotherap* or therap* or intervention* or treatment*)).ti,ab.
5	((group or systemic* or strategic* or paradoxical* or conjoint* or multisystemic* or ecosystem*) adj1 therap*).ti,ab.
6	(solution-focused* adj3 therap*).ti,ab.
7	or/4-6
8	and/3,7
9	cochrane database of systematic reviews.jn.
10	(search or MEDLINE or systematic review).tw.
11	meta analysis.pt.
12	or/9-11
13	12 not (exp animals/ not humans.sh.)
14	and/8,13
15	14 and (english or german).lg.
16	..l/ 15 yr=2012-Current

2. The Cochrane Library

- Cochrane Database of Systematic Reviews: Issue 12 of 12, December 2019

#	Searches
#1	MeSH descriptor: [Psychotherapy] explode all trees
#2	MeSH descriptor: [Stress, Psychological] this term only and with qualifier(s): [therapy - TH]
#3	psychotherap*:ti,ab
#4	#1 or #2 or #3
#5	(family near/3 (psychotherap* or therap* or intervention* or treatment*)):ti,ab
#6	(couple* near/1 (therap* or treatment*)):ti,ab
#7	((group or systemic* or strategic* or paradoxical* or conjoint* or marital* or multisystemic*) near/1 therap*):ti,ab
#8	(solution-focused* near/3 therap*):ti,ab
#9	#5 or #6 or #7 or #8
#10	#4 and #9 with Cochrane Library publication date Between Jan 2012 and Dec 2019, in Cochrane Reviews, Cochrane Protocols

- Cochrane Database of Systematic Reviews: Issue 7 of 12, July 2021

#	Searches
#1	MeSH descriptor: [Psychotherapy] explode all trees
#2	psychotherap*:ti,ab
#3	#1 or #2
#4	(family near/3 (psychotherap* or therap* or intervention* or treatment*)):ti,ab
#5	((group or systemic* or strategic* or paradoxical* or conjoint* or multisystemic* or ecosystem*) near/1 therap*):ti,ab
#6	(solution-focused* near/3 therap*):ti,ab
#7	#4 OR #5 OR #6
#8	#3 AND #7 with Cochrane Library publication date Between Jan 2012 and Jul 2021, in Cochrane Reviews, Cochrane Protocols

Search for primary studies

1. MEDLINE via Ovid

- Ovid MEDLINE(R) 1946 to July 11, 2022

Adoption of the following search filters:

- RCT: Lefebvre (4) – Cochrane Highly Sensitive Search Strategy for identifying randomized trials in MEDLINE: sensitivity-maximizing version (2008 revision)

#	Searches
1	exp psychotherapy/
2	psychotherap*.ti,ab.
3	or/1-2
4	(family adj3 (psychotherap* or therap* or intervention* or treatment*)).ti,ab.
5	((group or systemic* or strategic* or paradoxical* or conjoint* or multisystemic* or ecosystem*) adj1 therap*).ti,ab.
6	(solution-focused* adj3 therap*).ti,ab.
7	or/4-6
8	and/3,7
9	exp pediatrics/
10	(infan* or newborn* or new-born or perinat* or neonat* or baby or baby* or babies or toddler* or minors or minors* or boy or boys or boyfriend or boyhood or girl* or kid or kids or child or child* or children* or schoolchild* or schoolchild or adolescen* or juvenil* or youth* or teen* or under*age* or pubescen* or pediatric* or paediatric* or peadiatric* or prematur* or preterm*).af.
11	(school child or school child* or school or school*).ti,ab.
12	young adult/
13	((young adj2 (adult* or person* or individual* or people* or population* or man or men or wom#n)) or student*).ti,ab.
14	or/9-13
15	randomized controlled trial.pt.
16	controlled clinical trial.pt.
17	(randomized or placebo or randomly or trial or groups).ab.
18	drug therapy.fs.
19	or/15-18
20	19 not (exp animals/ not humans.sh.)
21	and/8,14,20
22	21 not (comment or editorial).pt.

- Ovid MEDLINE(R) Epub Ahead of Print and In-Process, In-Data-Review & Other Non-Indexed Citations July 11, 2022

#	Searches
1	psychotherap*.ti,ab.
2	(family adj3 (psychotherap* or therap* or intervention* or treatment*)).ti,ab.
3	((group or systemic* or strategic* or paradoxical* or conjoint* or multisystemic* or ecosystem*) adj1 therap*).ti,ab.
4	(solution-focused* adj3 therap*).ti,ab.
5	or/2-4
6	1 and 5
7	(infan* or newborn* or new-born or perinat* or neonat* or baby or baby* or babies or toddler* or minors or minors* or boy or boys or boyfriend or boyhood or girl* or kid or kids or child or child* or children* or schoolchild* or schoolchild or adolescen* or juvenil* or youth* or teen* or under*age* or pubescen* or pediatric* or paediatric* or peadiatric* or prematur* or preterm*).af.
8	(school child or school child* or school or school*).ti,ab.
9	((young adj2 (adult* or person* or individual* or people* or population* or man or men or wom#n)) or student*).ti,ab.
10	or/7-9
11	(clinical trial* or random* or placebo).ti,ab.
12	trial.ti.
13	or/11-12
14	and/6,10,13
15	14 not (comment or editorial).pt.

2. Embase via Ovid

- Embase 1974 to 2022 July 08

Adoption of the following search filters:

- RCT: Wong (3) – High sensitivity strategy

#	Searches
1	exp psychiatric treatment/
2	psychotherap*.ti,ab.
3	or/1-2
4	(family adj3 (psychotherap* or therap* or intervention* or treatment*)).ti,ab.
5	((group or systemic* or strategic* or paradoxical* or conjoint* or multisystemic* or ecosystem*) adj1 therap*).ti,ab.
6	(solution-focused* adj3 therap*).ti,ab.
7	or/4-6
8	and/3,7
9	exp pediatrics/
10	(infan* or newborn* or new-born or perinat* or neonat* or baby or baby* or babies or toddler* or minors or minors* or boy or boys or boyfriend or boyhood or girl* or kid or kids or child or child* or children* or schoolchild* or schoolchild or adolescen* or juvenil* or youth* or teen* or under*age* or pubescen* or pediatric* or paediatric* or peadiatric* or prematur* or preterm*).af.
11	(school child or school child* or school or school*).ti,ab.
12	young adult/
13	((young adj2 (adult* or person* or individual* or people* or population* or man or men or wom#n)) or student*).ti,ab.
14	or/9-11
15	random*.tw.
16	clinical trial*.mp.
17	exp health care quality/
18	or/15-17
19	18 not (exp animal/ not exp human/)
20	and/8,14,19
21	20 not medline.cr.
22	21 not (Conference Abstract or Conference Review or Editorial).pt.

3. PsycINFO via Ovid

- APA PsycINFO 1806 to July Week 1 2022

Adoption of the following search filters:

- RCT: Eady (5) – Combination of terms – small drop in specificity with a substantive gain in sensitivity

#	Searches
1	exp psychiatric treatment/
2	psychotherap*.ti,ab.
3	or/1-2
4	(family adj3 (psychotherap* or therap* or intervention* or treatment*)).ti,ab.
5	((group or systemic* or strategic* or paradoxical* or conjoint* or multisystemic* or ecosystem*) adj1 therap*).ti,ab.
6	(solution-focused* adj3 therap*).ti,ab.
7	or/4-6
8	and/3,7
9	exp pediatrics/
10	(infan* or newborn* or new-born or perinat* or neonat* or baby or baby* or babies or toddler* or minors or minors* or boy or boys or boyfriend or boyhood or girl* or kid or kids or child or child* or children* or schoolchild* or schoolchild or adolescen* or juvenil* or youth* or teen* or under*age* or pubescen* or pediatric* or paediatric* or peadiatric* or prematur* or preterm*).af.
11	(school child or school child* or school or school*).ti,ab.
12	young adult/
13	((young adj2 (adult* or person* or individual* or people* or population* or man or men or wom#n)) or student*).ti,ab.
14	or/9-11
15	random*.tw.
16	clinical trial*.mp.
17	exp health care quality/
18	or/15-17
19	18 not (exp animal/ not exp human/)
20	and/8,14,19
21	20 not medline.cr.
22	21 not (Conference Abstract or Conference Review or Editorial).pt.

4. The Cochrane Library via Wiley

- Cochrane Central Register of Controlled Trials: Issue 7 of 12, July 2022

#	Searches
#1	[mh "psychotherapy"]
#2	psychotherap*:ti,ab
#3	#1 or #2
#4	(family NEAR/3 (psychotherap* or therap* or intervention* or treatment*)):ti,ab
#5	((group or systemic* or strategic* or paradoxical* or conjoint* or multisystemic* or ecosystem*) NEAR/1 therap*):ti,ab
#6	(solution-focused* NEAR/3 therap*):ti,ab
#7	#4 or #5 or #6
#8	#3 and #7
#9	[mh "pediatrics"]
#10	infan* or newborn* or new-born or perinat* or neonat* or baby or baby* or babies or toddler* or minors or minors* or boy or boys or boyfriend or boyhood or girl* or kid or kids or child or child* or children* or schoolchild* or schoolchild or adolescen* or juvenil* or youth* or teen* or under*age* or pubescen* or pediatric* or paediatric* or peadiatric* or prematur* or preterm*
#11	(school child or school child* or school or school*):ti,ab
#12	[mh ^"young adult"]
#13	((young NEAR/2 (adult* or person* or individual* or people* or population* or man or men or wom#n)) or student*):ti,ab
#14	#9 or #10 or #11 or #12 or #13
#15	#8 AND #14 in Trials

A2.2 Study register

1. ClinicalTrials.gov

Provider: U.S. National Institutes of Health

- URL: http://www.clinicaltrials.gov
- Input interface: Expert Search

**Search strategy**
(EXPAND[Concept] "family therapy" OR EXPAND[Concept] "family intervention" OR EXPAND[Concept] "family treatment" OR EXPAND[Concept] "group therapy" OR EXPAND[Concept] "strategic therapy" OR EXPAND[Concept] "paradoxical therapy" OR EXPAND[Concept] "conjoint therapy" OR EXPAND[Concept] "solution-focused" OR “multisystemic therapy” OR psychotherapy AND (group OR family)) AND AREA[StdAge] EXPAND[Term] COVER[FullMatch] "Child"

2. International Clinical Trials Registry Platform Search Portal

Provider: World Health Organization

- URL: https://trialsearch.who.int
- Input interface: Standard Search

**Search strategy**
"family therapy" OR "family intervention" OR "family treatment" OR "family based" OR "group therapy" OR "strategic therapy" OR "paradoxical therapy" OR "conjoint therapy" OR "solution-focused" OR "multisystemic therapy" OR ((family OR group) AND psychotherapy) // Search for clinical trials in children

A3 Additional file 3 – Study pool of included RTCs

Table 2: Study pool of included RTCs (extended)

No.	Study	Full publication (in journals)	Register entry / result report from study registers
Class of mental disorder I: Affective disorders			
1	Brent 1997	yes (6-19)	no
Class of mental disorder II: Anxiety disorders and obsessive-compulsive disorders			
2	Lebowitz 2020	yes (20)	no
3	Peris 2013	yes (21-23)	yes (24) / no
4	Siqueland 2005	yes (25)	no
Class of mental disorder III: Eating disorders			
5	Le Grange 2015	yes (26-29)	yes (30) / no
6	Nyman-Carlsson 2019	yes (31, 32)	yes (33) / no
7	Schmidt 2007	yes (34-36)	yes (37) / no
Class of mental disorder IV: Hyperkinetic disorders			
8	Boyer 2015	yes (38-40)	yes (41) / no
Class of mental disorder V: Mental and behavioural disorders due to psychoactive substance use			
9	CYT	yes (42-48)	no
10	Dakof 2015	yes (49)	yes (50) / no
11	INCANT	yes (51-71)	yes[a] (72, 73) / no
12	Liddle 2008	yes (74-81)	no
13	Liddle 2018	yes (82)	yes (83) / no
14	Slesnick 2013	yes (84-88)	no
15	Waldron 2001	yes (89, 90)	no

a. Entries in the study registry for INCANT: ISRCTN51014277 for the total sample, ISRCTN00179361 for the Dutch sample.

A4 Additional file 4 – Characteristics on included RCTs

Table 3: Characteristics on included RCTs [alphabetical order] (multipage Table)

Class of mental disorder	No.	Study	▪ ST [n] vs. Comparator [n]	N total	Diagnosed disorders in %	Country	Patient relevant outcomes	Mean age (Range) in years	Sex f / m in %
I: Affective disorders	1	▪ Brent 1997 (6-19)	▪ Systemic Behaviour Family Therapy (SBFT) [n = 35] vs. Cognitive Behaviour Therapy (CBT) [n = 37]<br>▪ Study arm: Nondirective Supportive Treatment (not relevant for this publication) [n = 35]	▪ 107	▪ Major depression disorder (100)<br>▪ Anxiety diesorder (32)<br>▪ Dysthymic disorder (22)<br>▪ Disruptive disorder (21)<br>▪ Oppositional defiant disorder (16)	▪ USA	▪ Depressive symptoms<br>▪ Major depression<br>▪ Overall functioning<br>▪ Suicidal ideation	▪ 16 (13 – 18)	▪ 76 / 24

Table 3: Characteristics on included RCTs [alphabetical order] (multipage Table)

Class of mental disorder	No.	Study	▪ ST [n] vs. Comparator [n]	N total	Diagnosed disorders in %	Country	Patient relevant outcomes	Mean age (Range) in years	Sex f / m in %
II: Anxiety disorders and obsessive-compulsive disorders	2	▪ Lebowitz 2020 (20)	▪ Supportive Parenting for Anxious Childhood Emotions (SPACE) [n = 64] vs. Child-based Cognitive Behavioural Therapy (CBT) [n = 60]	▪ 124	▪ Any anxiety disorder (100)<br>▪ Generalised anxiety disorder (35)<br>▪ Social phobia (35)<br>▪ Seperation anxiety disorder (18)<br>▪ Specific phobia (12)	▪ USA	▪ Anxiety symptoms<br>▪ Overall improvement in clinical condition<br>▪ Overall severity of clinical condition<br>▪ Remission of anxiety disorder	▪ 10 (7 – 14)	▪ 53 / 47

Table 3: Characteristics on included RCTs [alphabetical order] (multipage Table)

Class of mental disorder	No.	Study	▪ ST [n] vs. Comparator [n]	N total	Diagnosed disorders in %	Country	Patient relevant outcomes	Mean age (Range) in years	Sex f / m in %
**II: Anxiety disorders and obsessive-compulsive disorders**	**3**	▪ Peris 2013 (21-24)	▪ Positive Family Interaction Therapy (PFIT) + Exposure and Response Prevention [n = 32] vs. Standard Treatment (Exposure and Response Prevention) [n = 30]	▪ 62	▪ Obsessive compulsive disorder (100)<br>▪ Anxiety disorder (inkl. separation, social, generalised, and specific phobia) (45)<br>▪ Depression (inkl. MDD and dysthymia) (15)<br>▪ Attention-deficit / hyper-activity disorder; (22)<br>▪ Oppositional defiant disorder (10)<br>▪ Chronic tic disorder / Tourette disorder (12)<br>▪ Autism-spectrum disorder (5)	▪ USA	▪ OCD symptoms<br>▪ Overall functioning<br>▪ Overall improvement in clinical condition	▪ 13 (8 – 17)	▪ 43 / 57

Table 3: Characteristics on included RCTs [alphabetical order] (multipage Table)

Class of mental disorder	No.	Study	▪ ST [n] vs. Comparator [n]	N total	Diagnosed disorders in %	Country	Patient relevant outcomes	Mean age (Range) in years	Sex f / m in %
II: Anxiety disorders and obsessive-compulsive disorders	4	▪ Siqueland 2005 (25)	▪ Attachment-based Family Therapy + Individual Cognitive-Behavioural Treatment (ABFT-CBT) [n = 5] vs. Individual Cognitive-Behavioural Treatment (CBT) [n = 6]	▪ 11	▪ Any anxiety disorder (100)<br>▪ Generalised anxiety disorder (91)<br>▪ Major depressive disorder (36)<br>▪ Social phobia (18)<br>▪ Separation anxiety disorder (9)<br>▪ Simple phobia (9)	▪ USA	▪ Anxiety symptoms<br>▪ Remission of anxiety disorder (primary diagnosis)	▪ 15 (12 – 18)	▪ 27 / 73
III: Eating disorders	5	▪ Le Grange 2015 (26-30)	▪ Family-based Treatment for Adolescent Bulimia nervosa (FBT) [n = 52] vs. Cognitive-Behavioural Therapy Adapted for Adolescents (CBT) [n = 58]<br><br>▪ Study arm: Nonspecific Intervention / Supportive Psychotherapy (not relevant for this publication) [n = 20]	▪ 130	▪ (partial) Bulimia nervosa (100)	▪ USA	▪ Binge eating and compensatory behaviours<br>▪ Body weight<br>▪ Hospitalisation<br>▪ Symptoms of bulimia nervosa	▪ 16 (12 – 18)	▪ 94 / 6

Table 3: Characteristics on included RCTs [alphabetical order] (multipage Table)

Class of mental disorder	No.	Study	▪ ST [n] vs. Comparator [n]	N total	Diagnosed disorders in %	Country	Patient relevant outcomes	Mean age (Range) in years	Sex f / m in %
III: Eating disorders	6	▪ Nyman-Carlsson 2019 (31-33)	▪ Family / Individual Therapy for Young Adults (FT-YA) [n = 40] vs. Cognitive Behavioural Therapy for Young Adults (CBT-YA) [n = 38]	▪ 78	▪ Anorexia nervosa (100)	▪ Sweden	▪ Eating disorder<br>▪ Body weight<br>▪ Hospitalisation<br>▪ Symptoms of anorexia nervosa	▪ 19 (17 – 24)	▪ 100 / 0
III: Eating disorders	7	▪ Schmidt 2007 (34-37)	▪ Family Therapy [n = 41] vs. Cognitive Behaviour Therapy Guided Self-Care (CBT Guided Self-Care) [n = 44]	▪ 85	▪ Bulimia nervosa (70)<br>▪ Eating disorder not otherwise specified (30)[i]	▪ UK	▪ Binge eating and compensatory behaviours<br>▪ Body weight	▪ 18 (13 – 20)	▪ 98 / 2
IV: Hyper-kinetic disorders	8	▪ Boyer 2015 (38-41)	▪ Solution-focused Treatment / Therapy (SFT) [n = 76] vs. Plan My Life (PML) [n = 83]	▪ 159	▪ Attention-deficit / hyper-activity disorder (100)<br>▪ Oppositional defiant disorder (63)<br>▪ Depression (40)<br>▪ Anxiety disorder (50)	▪ Netherlands	▪ Discontinuations due to adverse events<br>▪ Executive functioning<br>▪ Externalising problems<br>▪ Overall functioning	▪ 14 (12 – 17)	▪ 26 / 74

Table 3: Characteristics on included RCTs [alphabetical order] (multipage Table)

Class of mental disorder	No.	Study	▪ ST [n] vs. Comparator [n]	N total	Diagnosed disorders in %	Country	Patient relevant outcomes	Mean age (Range) in years	Sex f / m in %
V: Mental and behavioural disorders caused by psychotropic substances	9	▪ CYT (42-48)	▪ Multidimensional Family Therapy (MDFT) [n = 100] vs. Motivational Enhancement Therapy / Cognitive Behavioural Therapy [5 weeks] (MET/CBT5) [n = 102]<br>▪ Multidimensional Family Therapy (MDFT) [n = 100] vs. Adolescent Community Reinforcement Approach (ACRA) [n = 100]	▪ 600	▪ Cannabis abuse disorder (40)<br>▪ Cannabis dependence disorder (46)<br>▪ Alcohol abuse disorder / alcohol dependence disorder (37)<br>▪ Other drug abuse disorder / other drug dependence disorder (12)	▪ USA	▪ Substance use (any substance, self-report)	▪ 16 (12 – 18)	▪ 18 / 82

Table 3: Characteristics on included RCTs [alphabetical order] (multipage Table)

Class of mental disorder	No.	Study	▪ ST [n] vs. Comparator [n]	N total	Diagnosed disorders in %	Country	Patient relevant outcomes	Mean age (Range) in years	Sex f / m in %
V: Mental and behavioural disorders caused by psychotropic substances	10	▪ Dakof 2015 (49, 50)	▪ Multidimensional Family Therapy (MDFT) [n = 55] vs. Adolescent Group Therapy (AGT) [n = 57]	▪ 112	▪ Cannabis abuse disorder (61)<br>▪ Cannabis dependence disorder (30)<br>▪ Alcohol abuse disorder (4)<br>▪ Alcohol dependence abuse disorder (17)<br>▪ Other drug abuse disorder (7)<br>▪ Other drug dependence disorder (17)	▪ USA	▪ Substance use problem severity<br>▪ Externalising problems	▪ 16 (13 – 18)	▪ 11 / 89

Table 3: Characteristics on included RCTs [alphabetical order] (multipage Table)

Class of mental disorder	No.	Study	▪ ST [n] vs. Comparator [n]	N total	Diagnosed disorders in %	Country	Patient relevant outcomes	Mean age (Range) in years	Sex f / m in %
V: Mental and behavioural disorders caused by psychotropic substances	11	▪ INCANT (51-73)	▪ Multidimensional Family Therapy (MDFT) [n = 212] vs. Treatment as Usual (TAU) [n = 238]	▪ 450	▪ Cannabis abuse disorder (16)<br>▪ Cannabis dependence disorder (84)<br>▪ Alcohol abuse disorder / alcohol dependence disorder (40)<br>▪ Other drug abuse disorder / other drug dependence disorder (< 5)	▪ Belgium, France, Germany, Netherlands, Switzerland	▪ Cannabis use disorder<br>▪ Symptoms of cannabis use disorder<br>▪ Substance use detected by laboratory tests<br>▪ Substance use problem severity<br>▪ Use of cannabis<br>▪ Use of substances for which criteria for a substance use disorder are not met<br>▪ Externalising problems<br>▪ Internalising problems	▪ 16 (13 – 18)	▪ 15 / 85

Table 3: Characteristics on included RCTs [alphabetical order] (multipage Table)

Class of mental disorder	No.	Study	▪ ST [n] vs. Comparator [n]	N total	Diagnosed disorders in %	Country	Patient relevant outcomes	Mean age (Range) in years	Sex f / m in %
V: Mental and behavioural disorders caused by psychotropic substances	12	▪ Liddle 2008 (74-81)	▪ Multidimensional Family Therapy (MDFT) [n = 112] vs. Cognitive Behavioural Therapy (CBT) [n = 112]	▪ 224	▪ Cannabis abuse disorder (13)<br>▪ Cannabis dependence disorder (75)<br>▪ Alcohol abuse disorder (4)<br>▪ Alcohol dependence disorder (20)<br>▪ Other drug abuse disorder (2)<br>▪ Other drug dependence disorder (15)	▪ USA	▪ Substance use problem severity<br>▪ Use of cannabis<br>▪ Use of substances for which criteria for a substance use disorder are not met	▪ 15 (12 – 18)	▪ 19 / 81
V: Mental and behavioural disorders caused by psychotropic substances	13	▪ Liddle 2018 (82, 83)	▪ Multidimensional Family Therapy (MDFT) [n = 57] vs. Residential Treatment (RT) [n = 56]	▪ 113	▪ Cannabis use disorder (100)<br>▪ Alcohol use disorder (71)<br>▪ Opioid use disorder (33)	▪ USA	▪ Substance use problem severity<br>▪ Externalising problems<br>▪ Internalising problems	▪ 15 (13 – 18)	▪ 25 / 75

Table 3: Characteristics on included RCTs [alphabetical order] (multipage Table)

Class of mental disorder	No.	Study	ST [n] vs. Comparator [n]	N total	Diagnosed disorders in %	Country	Patient relevant outcomes	Mean age (Range) in years	Sex f / m in %
V: Mental and behavioural disorders caused by psychotropic substances	14	▪ Slesnick 2013 (84-88)	▪ Ecologically-Based Family Therapy (EBFT) [n = 61] vs. Community Reinforcement Approach (CRA) [n = 57]	▪ 179	▪ Not specified	▪ USA	▪ Use of substances for which criteria for a substance use disorder are not met<br>▪ Externalising problems<br>▪ Internalising problems	▪ 15 (12 – 17)	▪ 52,5 / 47,5
V: Mental and behavioural disorders caused by psychotropic substances	15	▪ Waldron 2001 (89, 90)	▪ Functional Family Therapy (FFT) [n = 30] vs. Individual Cognitive-Behavioural Therapy (CBT) [n = 31]	▪ 120	▪ Not specified	▪ USA	▪ Substance use detected by laboratory tests<br>▪ Use of cannabis<br>▪ Externalising problems<br>▪ Internalising problems	▪ 16 (13 – 17)	▪ 20 / 80

ABFT: Attachment-based Family Therapy; ACRA: Adolescent Community Reinforcement Approach; AGT: Adolescent Group Therapy; CBT: Cognitive Behaviour / Behavioural Therapy; CRA: Community Reinforcement Approach; DSM: Diagnostic and Statistical Manual of Mental Disorders; EBFT: Ecologically-Based Family Therapy; f: female; FBT: Family-based Treatment for Adolescent Bulimia nervosa; FFT: Functional Family Therapy; FT: Family / Individual Therapy; m: male; MDFT: Multidimensional Family Therapy; MET: Motivational Enhancement; N: number of randomized (or included) patients; OCD: Obsessive–compulsive disorder; PFIT: Positive Family Interaction Therapy; PML: Plan My Life; RCT: Randomized controlled Trial; RT: Residential Treatment; SBFT: Systemic Behaviour Family Therapy; SFT: Solution-focused Treatment / Therapy; SPACE: Supportive Parenting for Anxious Childhood Emotions; TAU: Treatment as Usual; vs.: versus, YA: Young Adults

A5 Additional file 5 – Risk of bias assessment

Table 4: Risk of bias assessment – study level

No.	Study	Generation of randomization sequence	Allocation concealment	Blinding		Result-independent reporting	Lack of other aspects	Risk of bias study level
				Patient	Treating staff			
1	Brent 1997 (6-19)	✓	?	–	–	–	–	High
2	Lebowitz 2020 (20)	✓	?	–	–	–	–	High
3	Peris 2013 (21-24)	✓	?	–	–	–	–	High
4	Siqueland 2005 (25)	?	?	–	–	–	–	High
5	Le Grange 2015 (26-30)	?	?	–	–	–	–	High
6	Nyman-Carlsson 2019 (31-33)	✓	?	–	–	–	–	High
7	Schmidt 2007 (34-37)	✓	✓	X	X	?	✓	Low
8	Boyer 2015 (38-41)	✓	✓	X	X	?	✓	Low
9	CYT (42-48)	✓	?	–	–	–	–	High
10	Dakof 2015 (49, 50)	✓	?	–	–	–	–	High
11	INCANT (51-73)	✓	✓	X	X	✓	✓	Low
12	Liddle 2008 (74-81)	✓	?	–	–	–	–	High
13	Liddle 2018 (82, 83)	✓	?	–	–	–	–	High
14	Slesnick 2013 (84-88)	✓	?	–	–	–	–	High
15	Waldron 2001 (89, 90)	?	?	–	–	–	–	High

–not rated; ✓ yes; X no; ? unclear

If the evaluation of the criteria "adequate generation of the randomization sequence" and / or "concealment of group assignment" revealed a high bias on study level, the risk of bias for all outcomes was rated as high and an evaluation of the remaining criteria was omitted. When an outcome was reported subjectively, there was automatically a high bias on outcome level. Only in case of a low risk of bias at study level, an outcome-specific assessment was performed (**Table 5**). A high risk of bias at the outcome level leads to moderate qualitative certainty of the results, a low risk of bias corresponds to a moderate risk of bias.

Table 5: Risk of bias assessment – outcome level

No.	Outcome	Study	Blinding outcome investigator	ITT principle adequately implemented	Result-independent reporting	Lack of other aspects	Risk of bias outcome level	Qualitative certainty of results
12	Binge eating and compensatory behaviours	Schmidt 2007 (34-37)	X	–	–	–	High	Moderate
13	Body weight	Schmidt 2007 (34-37)	✓	X	✓	✓	High	Moderate
18	Discontinuations due to adverse events	Boyer 2015 (38-41)	X	–	–	–	High	Moderate
19	Executive functioning	Boyer 2015 (38-41)	X	–	–	–	High	Moderate
20	Externalising problems	Boyer 2015 (38-41)	X	–	–	–	High	Moderate
21	Overall functioning	Boyer 2015 (38-41)	X	–	–	–	High	Moderate
22	Cannabis use disorder	INCANT (51-73)	X	–	–	–	High	Moderate
23	Externalising problems	INCANT (51-73)	X	–	–	–	High	Moderate
24	Internalising problems	INCANT (51-73)	X	–	–	–	High	Moderate
25	Symptoms of cannabis use disorder	INCANT (51-73)	X	–	–	–	High	Moderate
27	Substance use detected by laboratory tests	INCANT (51-73)	✓	✓	✓	✓	Low	High
28	Substance use problem severity	INCANT (51-73)	X	–	–	–	High	Moderate
29	Use of cannabis	INCANT (51-73)	X	–	–	–	High	Moderate
30	Use of substances for which criteria for a substance use disorder are not met	INCANT (51-73)	X	–	–	–	High	Moderate

– not rated; ✓ yes, X no

ITT: Intention to treat

A6 Additional file 6 – All results

A6.1 Tabular view – Class of mental disorders I: Affective disorders

Table 6: Affective disorders – results for all patient-relevant outcomes with assessable data (multipage Table)

No.	Outcome<br>Operationalisation<br>Length of follow-up	N<br>ST	N<br>COM	Effect<br>measure	Effect<br>estimate	[95 %-CI]	p-value	Direction of effect	Study	Conclusion on benefit at outcome level	Conclusion on benefit across outcomes
	**ST vs. COM**										↘[a]
**1**	**Depressive symptoms**									↘	
	**BDI**										
	Week 12 – 16	29	35	Hedges’ g	0.38[b]	[−0.12; 0.88][b]	0.130[b, c]	▼	Brent 1997 (6-19)		
	Month 26.8 – 27.7	n/a	n/a	Hedges’ g	n/a	n/a	> 0.05[d]	?	Brent 1997 (6-19)		
	**BDI < 9 (Proportion of patients who fell below a cut-off of 9 on the BDI on at least 3 consecutive therapy sessions (sustained until the end of therapy.))**										
	Week 12 – 16	29	35	OR	0.35[b]	[0.13; 0.97][b]	**0.045**[b, e]	▼	Brent 1997 (6-19)		
**2**	**Major depression**									↔	
	Diagnosis of major depressive disorder (responder analysis) (Kiddie-SADS)										
	Week 12 – 16	31	35	OR	2.30[b]	[0.72; 7.32][b]	0.173[b, e]	▼	Brent 1997 (6-19)		
**3**	**Overall functioning**									↔	
	**CGAS**										
	Week 12 – 16	31	35	Hedges’ g	−0.20[b]	[−0.69; 0.28][b]	0.408[b, c]	▼	Brent 1997 (6-19)		
	Month 26.8 – 27.7	n/a	n/a	Hedges’ g	n/a	n/a	> 0.05[d]	?	Brent 1997 (6-19)		
	**CGAS < 60**										
	Week 12 – 16	31	35	OR	1.59[b]	[0.55; 4.57][b]	0.530[b, e]	▲	Brent 1997 (6-19)		
	Month 26.8 – 27.7	n/a	n/a	OR	n/a	n/a	> 0.05[d]	?	Brent 1997 (6-19)		

Table 6: Affective disorders – results for all patient-relevant outcomes with assessable data (multipage Table)

No.	Outcome<br>Operationalisation<br>Length of follow-up	N ST	N COM	Effect measure	Effect estimate	[95 %-CI]	p-value	Direction of effect	Study	Conclusion on benefit at outcome level	Conclusion on benefit across outcomes
**4**	**Suicidal ideation**									↔	
	**Proportion of patients with currently existing suicidal ideation that was associated with a planned suicide or suicide attempt. K-SADS-P/E > 4 (unclear, K-SADS-P/E > 4 or K-SADS-P/E ≥ 4, criterion specified from Brent 1997 (6-19))**										
	Week 12 – 16	31	35	OR	0.74[b]	[0.11; 4.72][b]	0.775[b, e]	▲	Brent 1997 (6-19)		

a. Hint of a lesser benefit of ST compared to CBT across all outcomes based on the statistically significant effect to the disadvantage of ST for depressive symptoms (BDI < 9) along with the fact that the point estimates for other outcomes indicated a lower benefit of ST.
b. own calculation
c. t-Test
d. comparison of all 3 study arms
e. CSZ-Test (91)
↘: hint of less benefit of ST, one study
↔: no proof or indication or hint of a greater benefit or harm, one study
▲: effect in favour of ST
▼: effect to the disadvantage of ST
? Direction of effect is unclear.
significant in **bold** (p < 0.05)

BDI: Beck Depression Inventory; CBT: Cognitive Behaviour Therapy; CI: Confidence interval; CGAS: Children's Global Assessment Scale; COM: Comparator; K-SADS-P/E: Schedule for Affective Disorders and Schizophrenia for School-age Children, Present Episode and Epidemiologic versions; N: Number of analysed patients; n/a: not available or not specified in the study; OR: Odds Ratio; ST: Systemic Therapy; vs.: versus

A6.2 Tabular view – Class of mental disorders II: Anxiety disorders

Table 7: Anxiety disorders – results for all patient-relevant outcomes with assessable data (comparison 1: ST versus CBT) (multipage Table)

No.	Outcome<br>Operationalisation<br>Length of follow-up	N ST	N COM	Effect measure	Effect estimate	[95 %-CI]	p-value	Direction of effect	Study	Conclusion on benefit at outcome level	Conclusion on benefit across outcomes
	**ST vs. COM**										↔[a]
5	**Anxiety symptoms**									↔	
	**PARS**										
	Week 12	49	48	MD	−1.10[b]	[−2.82; 0.62][b]	0.207[b,c]	▲	Lebowitz 2020 (20)		
	**SCARED child report**										
	Week 12	49	48	MD	2.49[b]	[−3.14; 8.12][b]	0.382[b,c]	▼	Lebowitz 2020 (20)		
6	**Overall improvement in clinical condition**									↔	
	**CGI-I (Summary assessment of change between baseline and follow-up in terms of psychiatric symptomatology (frequency and intensity), experience of distress, and functional impairment in everyday life (work, school, relationships); clinician-assessed change in illness severity in relation to the specified dimensions between baseline survey and follow-up survey was recorded.)**										
	Week 12	49	48	Hedges' g	−0.06[b]	[−0.46; 0.34][b]	0.769[b, c]	▲	Lebowitz 2020 (20)		
	**CGI-I ≤ 2**										
	Week 12	48	49	OR	2.27[b]	[0.77; 6.65][b]	0.7[b, d]	▲	Lebowitz 2020 (20)		
7	**Overall severity of clinical condition**									↔	
	**CGI-S (Summary assessment of psychiatric symptoms (frequency and intensity), stress experience, and functional impairment in everyday life (work, school, relationships); average severity over the past 7 days was recorded.)**										
	Week 12	49	48	Hedges' g	−0.15[b]	[−0.55; 0.25][b]	0.464[b, c]	▲	Lebowitz 2020 (20)		
	**CGI-I ≤ 2**										
	Week 12	48	49	OR	0.97[b]	[0.43; 2.17][b]	0.88[b, d]	▼	Lebowitz 2020 (20)		

Table 7: Anxiety disorders – results for all patient-relevant outcomes with assessable data (comparison 1: ST versus CBT) (multipage Table)

No.	Outcome<br>Operationalisation<br>Length of follow-up	N<br>ST	N<br>COM	Effect<br>measure	Effect<br>estimate	[95 %-CI]	p-value	Direction of effect	Study	Conclusion on benefit at outcome level	Conclusion on benefit across outcomes
8	**Remission of anxiety disorder**									↔	
	**ADIS-c/pa (Number of patients who no longer met the required diagnostic criteria according to DSM-IV for any anxiety disorder; diagnosis by means of ADIS-c/p)**										
	Week 12	n/a	n/a	n/a	n/a	n/a	0.57[e]	▲	Lebowitz 2020 (20)		

a. No proof or indication or hint of a greater benefit or harm of ST across all outcomes based on effects which were statistically not significant.
b. own calculation
c. t-Test
d. Chi-square test
↔: no proof or indication or hint of a greater benefit or harm, one study
▲: effect in favour of ST
▼: effect to the disadvantage of ST
significant in **bold** ($p < 0.05$)

ADIS-c/p: Anxiety Disorders Interview Schedule for DSM-IV, child and parent version; CGI-I: Clinical Global Impressions – Improvement; CGI-S: Clinical Global Impressions – Severity; CI: Confidence interval; COM: Comparator; DSM: Diagnostic and Statistical Manual of Mental Disorders; MD: Mean difference; N: Number of analysed patients; n/a: not available or not specified in the study; OR: Odds Ratio; PARS: Pediatric Anxiety Rating Scale; SCARED: Screen for Childhood Anxiety Related Emotional Disorders; ST: Systemic Therapy; vs.: versus

A6.3 Tabular view – Class of mental disorders II: Anxiety disorders and obsessive-compulsive disorders

Table 8: Anxiety disorders and obsessive-compulsive disorders – results for all patient-relevant outcomes with assessable data (comparison 2: ST as an add-on to CBT versus CBT alone) (multipage Table)

No.	Outcome / Operationalisation / Length of follow-up	N ST	N COM	Effect measure	Effect estimate	[95 %-CI]	p-value	Direction of effect	Study	Conclusion on benefit at outcome level	Conclusion on benefit across outcomes
	**ST vs. COM**										↗[a]
5	**Anxiety symptoms**									↔	
	**BAI**										
	Month 3.7	5	6	MD	−1.70[b]	[−13.47; 10.07][b]	0.751[b]	▲	Siqueland 2005 (25)		
	Month 10 – 13	5	6	MD	1.80[b]	[-4.28; 7.88][b]	0.520[b]	▼	Siqueland 2005 (25)		
	**HAM-A**										
	Month 3.7	5	6	MD	−2.90[b]	[−10.41; 4.61][b]	0.405[b]	▲	Siqueland 2005 (25)		
	Month 10 – 13	5	6	MD	−0.40[b]	[−7.22; 6.42][b]	0.897[b]	▲	Siqueland 2005 (25)		
6	**Overall improvement in clinical condition**									↗	
	**CGI-I (Summary assessment of change between baseline and follow-up in terms of psychiatric symptomatology (frequency and intensity), experience of distress, and functional impairment in everyday life (work, school, relationships); clinician-assessed change in illness severity in relation to the specified dimensions between baseline survey and follow-up survey was recorded.)**										
	Week 14	31	30	Hedges' g	−0.64[b]	[−1.15; −0.12][b]	**0.015**[b, c]	▲	Peris 2013 (21-24)		
	**CGI-I ≤ 2**										
	Week 14	31	30	OR	3.15[b]	[1.10; 8.99][b]	**0.03**[b, d]	▲	Peris 2013 (21-24)		
9	**Obsessive–compulsive disorder (OCD) symptoms**									↗	
	**CY-BOCS**										
	Week 14	31	30	Hedges' g	−0.54[b]	[−1.05; −0.02][b]	**0.038**[e]	▲	Peris 2013 (21-24)		
	**CY-BOCS ≤ 14 (Proportion of patients who fell below a cut-off of 15 on the CY-BOCS.)**										
	Week 14	31	30	OR	3.81[b]	[1.29; 11.20][b]	**0.01**[d]	▲	Peris 2013 (21-24)		

Table 8: Anxiety disorders and obsessive-compulsive disorders – results for all patient-relevant outcomes with assessable data (comparison 2: ST as an add-on to CBT versus CBT alone) (multipage Table)

No.	Outcome<br>Operationalisation<br>Length of follow-up	N ST	N COM	Effect measure	Effect estimate	[95 %-CI]	p-value	Direction of effect	Study	Conclusion on benefit at outcome level	Conclusion on benefit across outcomes
**10**	**Overall functioning**									↗	
	**Overall functioning (not disease-specific)**										
	**CGAS**										
	Week 14	10[f]	10[f]	Hedges' g	0.56[b]	[−0.34; 1.46][b]	0.207[b, c]	▲	Peris 2013 (21-24)		
	**Overall functioning (specific for obsessive-compulsive disorder)**										
	**COIS-R**										
	Week 14	31	30	Hedges' g	−0.75[b]	[−1.27; −0.23][b]	**0.004**[b, e]	▲	Peris 2013 (21-24)		
**11**	**Remission of anxiety disorder (primary diagnosis)**									↔	
	**ADIS-Child (Number of patients who no longer met all diagnostic criteria for the primary anxiety disorder in each case; most patients met criteria for generalised anxiety disorder according to DSM-IV at baseline; diagnosis by ADIS-Child)**										
	Month 3.7	5	6	OR	0.33[b]	[0.03; 1.26][b]	0.522[b]	▼	Siqueland 2005 (25)		
	Month 10 – 13	5	6	OR	0.23[b]	[0.01; 7.05][b]	0.325[b]	▼	Siqueland 2005 (25)		

Table 8: Anxiety disorders and obsessive-compulsive disorders – results for all patient-relevant outcomes with assessable data (comparison 2: ST as an add-on to CBT versus CBT alone) (multipage Table)

No.	Outcome<br>Operationalisation<br>Length of follow-up	N<br>ST	N<br>COM	Effect<br>measure	Effect<br>estimate	[95 %-CI]	p-value	Direction of effect	Study	Conclusion on benefit at outcome level	Conclusion on benefit across outcomes

a. Hint of greater benefit of ST as an add-on to CBT compared with CBT alone across all outcomes based on the statistically significant effects in favour of ST as an add-on to CBT for obsessive–compulsive disorder symptoms (CY-BOCS ≤ 14), overall functioning (COIS-R), and overall improvement in clinical condition (CGI-I ≤ 2).
b. own calculation
c. t-Test
d. Chi-square test
e. Linear Mixed Effects Regression Model
f. Data on CGAS were only available for a subsample from Peris 2013 (21-24)
↗: hint of a greater benefit of ST, one study
↔: no proof or indication or hint of a greater benefit or harm, one study
▲: effect in favour of ST
▼: effect to the disadvantage of ST
significant in **bold** ($p < 0.05$)

ADIS-c/p: Anxiety Disorders Interview Schedule for DSM-IV, child and parent version; BAI: Beck Anxiety Inventory; CGAS: Children's Global Assessment Scale; CGI-I: Clinical Global Impressions – Improvement; CI: Confidence interval; COM: Comparator; COIS-R: Child Obsessive Compulsive Impact Scale-Revised; CY-BOCS: Children's Yale-Brown Obsessive Compulsive Scale; DSM: Diagnostic and Statistical Manual of Mental Disorders; HAM-A: Hamilton Anxiety Rating Scale; MD: Mean difference; N: Number of analysed patients; OR: Odds Ratio; ST: Systemic Therapy; vs.: versus

A6.4 Tabular view – Class of mental disorders III: Eating disorders

Table 9: Eating disorders – results for all patient-relevant outcomes with assessable data (multipage Table)

No.	Outcome / Operationalisation / Length of follow-up	N ST	N COM	Effect measure	Effect estimate	[95 %-CI]	p-value	Direction of effect	Study	Conclusion on benefit at outcome level	Conclusion on benefit across outcomes
	**ST vs. COM**										↔[a]
12	**Binge eating and compensatory behaviours**									↔	
	**Eating episodes in bulimia nervosa (number of episodes in the last 4 weeks)**										
	Month 6.4	43	43	MD	−3.70[b]	[−10.60; 3.20][b]	0.289[b]	▲	Le Grange 2015 (26-30)		
	**Application of compensatory behaviours in bulimia nervosa (number of episodes)**										
	Month 6.4	43	43	MD	−5.60[b]	[−12.91; 1.71][b]	0.13[b]	▲	Le Grange 2015 (26-30)		
	**Eating episodes in bulimia nervosa (number of episodes)**										
	Month 0 – 12	n/a	n/a	n/a	n/a	n/a	0.21[c]	?	Schmidt 2007 (34-37)		
	**Episodes of vomiting episodes in bulimia nervosa (number of episodes); average number of episodes per week in the last 28 days**										
	Month 0 – 12	n/a	n/a	n/a	n/a	n/a	0.20[c]	?	Schmidt 2007 (34-37)		
	**No binge eating and compensatory behaviours (in bulimia nervosa)**										
	Month 6.4	51	58	RD	0.197[e]	n/a	**0.040**[e]	▲	Le Grange 2015 (26-30)		
	Month 12.4	51	58	RD	0.185[e]	n/a	**0.030**[e]	▲	Le Grange 2015 (26-30)		
	Month 18.4	51	58	RD	0.165[e]	n/a.	0.130[e]	▲	Le Grange 2015 (26-30)		
	**Binge eating (in bulimia nervosa)**										
	Month 6	32	31	OR	0.33[b,f]	[0.13; 0.86][b,f]	**0.023**[b,f]	▼	Schmidt 2007 (34-37)		
	**Compensatory behaviours in bulimia nervosa (vomiting)**										
	Month 6	32	31	OR	0.75[b,f]	[0.30; 1.87][b,f]	0.537[b,f]	▲	Schmidt 2007 (34-37)		

Table 9: Eating disorders – results for all patient-relevant outcomes with assessable data (multipage Table)

No.	Outcome<br>Operationalisation<br>Length of follow-up	N ST	N COM	Effect measure	Effect estimate	[95 %-CI]	p-value	Direction of effect	Study	Conclusion on benefit at outcome level	Conclusion on benefit across outcomes
	**Binge eating and compensatory behaviours (in bulimia nervosa)**										
	Month 6	32	31	OR	0.63[b,f]	[0.25; 1.61][b,f]	0.330[b,f]	▲	Schmidt 2007 (34-37)		
	**reduced food intake (diet); (average number of days per week in the past 28 days) in bulimia nervosa**										
	Month 6	32	31	MD	0.30[b]	[−1.21; 1.81][b]	0.693[b, g]	▼	Schmidt 2007 (34-37)		
	**reduced food intake (fasting); (average number of days per week in the past 28 days) in bulimia nervosa**										
	Month 6	32	31	MD	0.10[b]	[−0.78; 0.98][b]	0.822[b, g]	▼	Schmidt 2007 (34-37)		
**13**	**Body weight**									↔	
	**Body weight in anorexia nervosa (BMI)**										
	Month 18	37	37	MD	0.28	[−0.51; 1.06]	0.49	▼	Nyman-Carlsson 2019 (31-33)		
	Month 36	37	37	MD	−0.64	[−1.43; 0.15]	0.11	▲	Nyman-Carlsson 2019 (31-33)		
**14**	**Eating disorder**									↔	
	**Diagnosis of anorexia nervosa**										
	Month 18	37	37	OR	1.00	[0.19; 5.31][b]	> 0.999[b, h]	▼	Nyman-Carlsson 2019 (31-33)		
	Month 36	37	37	OR	0.19	[0.01; 4.08][b]	0.207[b, h]	▲	Nyman-Carlsson 2019 (31-33)		
	**Diagnosis of bulimia nervosa**										
	Month 18	37	37	OR	0.32	[0.01; 8.23][b]	0.528[b, h]	▲	Nyman-Carlsson 2019 (31-33)		
	Month 36	37	37	OR	5.28	[0.24; 113.87][b]	0.207[b, h]	▼	Nyman-Carlsson 2019 (31-33)		

Table 9: Eating disorders – results for all patient-relevant outcomes with assessable data (multipage Table)

No.	Outcome<br>Operationalisation<br>Length of follow-up	N ST	N COM	Effect measure	Effect estimate	[95 %-CI]	p-value	Direction of effect	Study	Conclusion on benefit at outcome level	Conclusion on benefit across outcomes
	**Diagnosis of an unspecified eating disorder**										
	Month 18	37	37	OR	1.24	[0.34; 4.48][b]	0.834[b, h]	▼	Nyman-Carlsson 2019 (31-33)		
	Month 36	37	37	OR	2.73	[0.50; 15.09][b]	0.269[b, h]	▼	Nyman-Carlsson 2019 (31-33)		
	**No diagnosis**										
	Month 18	37	37	OR	1.00	[0.35; 2.89][b]	> 0.999[b, h]	▲	Nyman-Carlsson 2019 (31-33)		
	Month 36	37	37	OR	0.52	[0.14; 1.95][b]	0.528[b, h]	▼	Nyman-Carlsson 2019 (31-33)		
**15**	**Hospitalisation**									↔	
	**hospitalised patients**										
	Week 0 – 8	51	58	OR	0.11[b]	[0.01; 0.89][b]	**0.016**[b, h]	▲	Le Grange 2015 (26-30)		
	Month 0 – 6.4	51	58	OR	0.08[b]	[0.01; 0.61][b]	**0.015**	▲	Le Grange 2015 (26-30)		
	Month 0 – 18	37	37	OR	0.86[b]	[0.29; 2.54][b]	0.867[b, h]	▲	Nyman-Carlsson 2019 (31-33)		
**16**	**Symptoms of anorexia nervosa**									↔	
	**EDI-3 GPMC (for anorexia nervosa); Subscale General Psychological Maladjustment Composite of the Eating Disorder Inventory to detect psychological symptoms relevant to eating disorders.**										
	Month 18	37	37	MD	−0.40[c]	[−17.06; 16.20][c]	0.96[c]	▼	Nyman-Carlsson 2019 (31-33)		
	Month 36	37	37	MD	2.80[c]	[−13.81; 19.50][c]	0.74[c]	▲	Nyman-Carlsson 2019 (31-33)		

Table 9: Eating disorders – results for all patient-relevant outcomes with assessable data (multipage Table)

No.	Outcome<br>Operationalisation<br>Length of follow-up	N ST	N COM	Effect measure	Effect estimate	[95 %-CI]	p-value	Direction of effect	Study	Conclusion on benefit at outcome level	Conclusion on benefit across outcomes
	**EDI-3 EDRC (for anorexia nervosa); Subscale Eating Disorder Risk Composite of the Eating Disorder Inventory to detect symptoms of eating disorders.**										
	Month 18	37	37	MD	0.10[c]	[−9.46; 9.56]	0.99	▲	Nyman-Carlsson 2019 (31-33)		
	Month 36	37	37	MD	1.40[c]	[−8.13; 10.89]	0.78	▲	Nyman-Carlsson 2019 (31-33)		
	**Eating Disorder Index (for anorexia nervosa)**										
	Month 18	37	37	MD	0.05[c]	[−0.06; 0.16]	0.36	▲	Nyman-Carlsson 2019 (31-33)		
	Month 36	37	37	MD	0.05[c]	[−0.09; 0.13]	0.74	▲	Nyman-Carlsson 2019 (31-33)		
**17**	**Symptoms of bulimia nervosa**									↔	
	**global eating disorder symptomatology in bulimia nervosa (EDE global)**										
	Month 6.4	51	58	Cohen's d	0.223[e]	[−0.12; 0.56][b]	0.199[e]	▼	Le Grange 2015 (26-30)		
	Month 12.4	51	58	Cohen's d	0.142[e]	[−0.27; 0.55][b]	0.495[e]	▼	Le Grange 2015 (26-30)		
	Month 18.4	51	58	Cohen's d	0.309[e]	[−0.08; 0.70][b]	0.119[e]	▼	Le Grange 2015 (26-30)		
	**YBC total score (for bulimia nervosa)**										
	Month 6.4	51	58	Cohen's d	0.291[e]	[−0.06; 0.64][b]	0.100[e]	▼	Le Grange 2015 (26-30)		
	Month 12.4	51	58	Cohen's d	0.242[e]	[−0.13; 0.62][b]	0.204[e]	▼	Le Grange 2015 (26-30)		
	Month 18.4	51	58	Cohen's d	0.289[e]	[−0.09; 0.67][b]	0.133[e]	▼	Le Grange 2015 (26-30)		

Table 9: Eating disorders – results for all patient-relevant outcomes with assessable data (multipage Table)

No.	Outcome<br>Operationalisation<br>Length of follow-up	N<br>ST	N<br>COM	Effect<br>measure	Effect<br>estimate	[95 %-CI]	p-value	Direction of effect	Study	Conclusion on benefit at outcome level	Conclusion on benefit across outcomes

a. No proof or indication or hint of a greater benefit or harm of ST across all outcomes based on the lack of unidirectional effects for hospitalisation, an inconsistent data situation for binge eating and compensatory behaviours, and statistically not significant effects for the remaining outcomes.
b. own calculation
c. Repeated measurements model
d. Linear Mixed Effects Regression Model
e. Longitudinal Mixed Effects Model
f. Proportional Odds Model (categories: abstinent / subclinical / clinical)
g. t-test
h. CSZ-Test (91)
↔: no proof hint or indication of a greater benefit or harm, one study
▲: effect in favour of ST
▼: effect to the disadvantage of ST
?: Direction of effect is unclear.
significant in **bold** (p < 0.05)

BMI: Body-Mass-Index; CI: Confidence interval; COM: Comparator; EDE: Eating Disorder Examination; EDI: Eating Disorder Inventory; EDRC: EDI-3 Eating Disorder Risk Composite; GPMC: General Psychological Maladjustment Composite; MD: Mean difference; N: Number of analysed patients; n/a: not available or not specified in the study; OR: Odds Ratio; RD: Risik Difference, ST: Systemic Therapy; vs.: versus; YBC: Yale-Brown-Cornell Eating Disorder Scale

A6.5 Forest plots – Class of mental disorders III: Eating disorders

Systemic therapy (ST) vs. Cognitive behavioural therapy (CBT)
Binge eating and compensatory behaviours, 6 months
Fixed effect model - Mantel-Haenszel (for presentation of the weights)

Study	ST n/N	CBT n/N	OR (95% CI)	weight	OR	95% CI
Le Grange 2015	17/43	8/43		36.0	2.65	[1.01, 6.98]
Schmidt 2007	8/32	12/31		64.0	0.53	[0.18, 1.55]

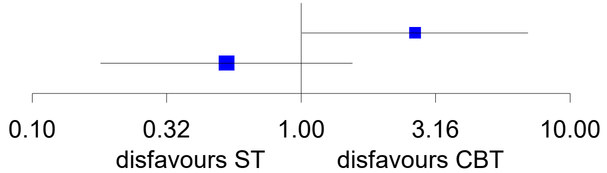

Heterogeneity: Q=4.76, df=1, p=0.029, I²=79.0%

Figure 1: Forest Plot for binge eating and compensatory behaviours after 6 months; Systemic Therapy (ST) vs. Cognitive Behavioural Therapy (CBT); Eating disorders

A6.6 Tabular view – Class of mental disorders IV: Hyperkinetic disorders

Table 10: Hyperkinetic disorders – results for all patient-relevant outcomes with assessable data (multipage Table)

No.	Outcome / Operationalisation / Length of follow-up	N ST	N COM	Effect measure	Effect estimate	[95 %-CI]	p-value	Direction of effect	Study	Conclusion on benefit at outcome level	Conclusion on benefit across outcomes
	**ST vs. COM**										↔[a]
**18**	**Discontinuations due to adverse events**									↔	
	**Termination due to suicidal ideation**										
	Month 2.1	67	79	OR	0.39[b]	[0.02; 9.67][b]	0.515[b, c]	▲	Boyer 2015 (38-41)		
**19**	**Executive functioning**									↔	
	**BRIEF, total score Global Executive Composite**										
	Month 2.1	67	79	Hedges' g	0.25[b]	[−0.08; 0.57][b]	0.137[b, d]	▼	Boyer 2015 (38-41)		
	Month 5.1	59	77	Hedges' g	0.11[b]	[−0.23; 0.45][b]	0.532[b, d]	▼	Boyer 2015 (38-41)		
	**BRIEF-Subscale plan / organize**										
	Month 2.1	67	79	Hedges' g	0.36[b]	[0.03; 0.69][b]	0.031[b, d]	▼	Boyer 2015 (38-41)		
	Month 5.1	59	77	Hedges' g	0.35[b]	[0.01; 0.69][b]	**0.043**[b, d]	▼	Boyer 2015 (38-41)		
	**D-KEFS Tower test (neuropsychological Test)**										
	Month 2.1	67	79	Hedges' g	0.05[b]	[−0.28; 0.37][b]	0.775[b, d]	▲	Boyer 2015 (38-41)		
	Month 5.1	59	77	Hedges' g	0.00[b]	[−0.34; 0.34][b]	0.988[b, d]	?	Boyer 2015 (38-41)		
	**D-KEFS TMT (neuropsychological Test)**										
	Month 2.1	67	79	Hedges' g	−0.02[b]	[−0.34; 0.31][b]	0.918[b, d]	▲	Boyer 2015 (38-41)		
	Month 5.1	59	77	Hedges' g	−0.04[b]	[−0.38; 0.30][b]	0.814[b, d]	▲	Boyer 2015 (38-41)		
	**BADS Key search (neuropsychological Test)**										
	Month 2.1	67	79	Hedges' g	0.03[b]	[−0.30; 0.36][b]	0.857[b, d]	▲	Boyer 2015 (38-41)		
	Month 5.1	59	77	Hedges' g	0.23[b]	[−0.11; 0.57][b]	0.181[b, d]	▲	Boyer 2015 (38-41)		

Table 10: Hyperkinetic disorders – results for all patient-relevant outcomes with assessable data (multipage Table)

No.	Outcome<br>Operationalisation<br>Length of follow-up	N ST	N COM	Effect measure	Effect estimate	[95 %-CI]	p-value	Direction of effect	Study	Conclusion on benefit at outcome level	Conclusion on benefit across outcomes
	**BADS Zoo map (neuropsychological Test)**										
	Month 2.1	67	79	Hedges' g	−0.27[b]	[−0.60; 0.06][b]	0.105[b, d]	▲	Boyer 2015 (38-41)		
	Month 5.1	59	77	Hedges' g	0.00[b]	[−0.34; 0.34][b]	> 0.999[b, d]	?	Boyer 2015 (38-41)		
**20**	**Externalising problems**									↔	
	**CBCL, externalising problems scale**										
	Month 2.1	67	79	Hedges' g	0.13[b]	[−0.19; 0.46][b]	0.424[b, d]	▼	Boyer 2015 (38-41)		
	Month 5.1	59	77	Hedges' g	−0.15[b]	[−0.49; 0.19][b]	0.377[b, d]	▲	Boyer 2015 (38-41)		
**21**	**Overall functioning**									↔	
	**Homework Problems Checklist**										
	Month 2.1	67	79	Hedges' g	0.36[b]	[0.04; 0.69][b]	**0.029**[b, d]	▼	Boyer 2015 (38-41)		
	Month 5.1	59	77	Hedges' g	0.12[b]	[−0.22; 0.45][b]	0.504[b, d]	▼	Boyer 2015 (38-41)		
	**IRS**										
	Month 2.1	67	79	Hedges' g	0.11[b]	[−0.21; 0.44][b]	0.498[b, d]	▼	Boyer 2015 (38-41)		
	Month 5.1	59	77	Hedges' g	0.03[b]	[−0.31; 0.37][b]	0.869[b, d]	▼	Boyer 2015 (38-41)		

Table 10: Hyperkinetic disorders – results for all patient-relevant outcomes with assessable data (multipage Table)

No.	Outcome<br>Operationalisation<br>Length of follow-up	N ST	N COM	Effect measure	Effect estimate	[95 %-CI]	p-value	Direction of effect	Study	Conclusion on benefit at outcome level	Conclusion on benefit across outcomes

a. No proof or indication or hint of a greater benefit or harm of ST across all outcomes based on effects which were either statistically not significant or were classified as not clinically relevant.
b. own calculation
c. CSZ-Test (91)
d. t-Test
↔: no proof or indication or hint of a greater benefit or harm, one study
▲: effect in favour of ST
▼: effect to the disadvantage of ST
?: Direction of effect is unclear.
significant in **bold** ($p < 0.05$)

BADS: Behavioural Assessment of the Dysexecutive Syndrome; BRIEF: Behaviour Rating Inventory of Executive Function; CBCL: Child Behaviour CheckList; CI: Confidence interval; COM: Comparator; D-KEFS: Delis-Kaplan Executive Function System; IRS: Impairment Rating Scale; ST: Systemic Therapy; N: Number of analysed patients; n/a: not available or not specified in the study; OR: Odds Ratio; TMT: Trail Making Test; vs.: versus

A6.7 Tabular view – Class of mental disorders V: Mental and behavioural disorders due to psychoactive substance use

Table 11: Mental and behavioural disorders due to psychoactive substance use – results for all patient-relevant outcomes with assessable data (multipage Table)

No.	Outcome<br>Operationalisation<br>Length of follow-up	N ST	N COM	Effect measure	Effect estimate	[95 %-CI]	p-value	Direction of effect	Study	Conclusion on benefit at outcome level	Conclusion on benefit across outcomes
	**ST vs. COM**										↗[a]
22	**Cannabis use disorder**									↗	
	**ADI-Light for Cannabis, Diagnosis of cannabis use disorder, specialised interview to detect symptoms of cannabis use disorder according to DSM-IV**										
	Month 12	212	238	OR[b]	1.68[c]	[1.15; 2.44][c]	**0.007**[c]	▲	INCANT (51-73)		
23	**Externalising problems**									⇑⇓	
	**Externalising subscale of YSR**										
	Month 0 – 2	57	56	n/a	n/a	n/a	≥ 0.05[d]	?	Liddle 2018 (82, 83)		
	Month 2	57	55	Hedges' g	−0.18	[−0.55; 0.19][c]	0.341[c, e]	▲	Liddle 2018 (82, 83)		
	Month 3	49	46	Hedges' g	0.11[c]	[−0.29; 0.51][c]	0.598[c, e]	▼	Slesnick 2013 (84-88)		
	Month 4	55	54	MD	0.82[c]	[−3.37; 5.01][c]	0.699[c, e]	▼	Liddle 2018 (82, 83)		
	Month 6	54	52	MD	1.07[c]	[−2.93; 5.07][c]	0.597[c, e]	▼	Dakof 2015 (49, 50)		
		163[f]	183[f]	MD	−0.04[c]	[−1.93; 1.85][c]	0.967[c, e]	▲	INCANT[g] (51-73)		
		42	42	MD	−2.18[c]	[−7.21; 2.85][c]	0.391[c, e]	▲	Slesnick 2013 (84-88)		
	Month 9	47	40	Hedges' g	−0.07[c]	[−0.50; 0.35][c]	0.726[c, e]	▲	Slesnick 2013 (84-88)		
	Month 12	46	45	MD	−0.53[c]	[−4.24; 3.18][c]	0.777[c, e]	▲	Dakof 2015 (49, 50)		
		56	53	MD	−0.64[c]	[−4.58; 3.30][c]	0.748[c]	▲	Liddle 2018 (82, 83)		
		180[f]	202[f]	MD	−0.48[c]	[−2.28; 1.32][c]	0.600[c, e]	▲	INCANT[g] (51-73)		
		58	60	n/a	n/a	n/a	0.401	?	INCANT[h] (51-73)		
		46	39	MD	1.72[c]	[−2.43; 5.87][c]	0.412[c]	▼	Slesnick 2013 (84-88)		

Table 11: Mental and behavioural disorders due to psychoactive substance use – results for all patient-relevant outcomes with assessable data (multipage Table)

No.	Outcome<br>Operationalisation<br>Length of follow-up	N ST	N COM	Effect measure	Effect estimate	[95 %-CI]	p-value	Direction of effect	Study	Conclusion on benefit at outcome level	Conclusion on benefit across outcomes
	Month 18	47	46	MD	0.88[c]	[−2.77; 4.53][c]	0.633[c, e]	▼	Dakof 2015 (49, 50)		
		57	55	MD	−2.01[c]	[−5.73; 1.71][c]	0.286[c]	▲	Liddle 2018 (82, 83)		
		45	41	MD	0.35[c]	[−4.07; 4.77][c]	0.875[c]	▼	Slesnick 2013 (84-88)		
	Month 2 – 18	57	56	n/a	n/a	n/a	≥ 0.05[d]	?	Liddle 2018 (82, 83)		
	Month 24	51	43	MD	−1.82[c]	[−5.38; 1.74][c]	0.313[c, e]	▲	Dakof 2015 (49, 50)		
		43	41	MD	−1.77[c]	[−6.11; 2.57][c]	0.419[c, e]	▲	Slesnick 2013 (84-88)		
	Month 0 – 24	n/a	n/a	n/a	n/a	n/a	≥ 0.05[i]	?	Slesnick 2013 (84-88)		
	Month 7 – 24	55	57	Cohen's d	0.39[d]	[0.02; 0.76][c]	**0.039**[d]	▲	Dakof 2015 (49, 50)		
	**Externalising subscale, parent / primary caretaker version of YSR, CBCL**										
	Month 3	49	47	Hedges' g	−0.18[c]	[−0.58; 0.22][c]	0.367[c, e]	▲	Slesnick 2013 (84-88)		
	Month 4	n/a	n/a	n/a	n/a	n/a	> 0.05	n/a	Waldron 2001 (89, 90)		
	Month 6	161[f]	180[f]	MD	−0.32[c]	[−2.80; 2.16][c]	0.800[c, e]	▲	INCANT[g] (51-73)		
		42	43	MD	−6.11[c]	[−12.20; −0.02][c]	**0.049**[c, e]	▲	Slesnick 2013 (84-88)		
	Month 7	n/a	n/a	n/a	n/a	n/a	> 0.05	n/a	Waldron 2001 (89, 90)		
	Month 9	46	41	Hedges' g	−0.11[c]	[−0.54; 0.31][c]	0.593[c, e]	▲	Slesnick 2013 (84-88)		
	Month 12	171[f]	193[f]	MD	0.99[c]	[−1.17; 3.15][c]	0.368[c, e]	▼	INCANT[g] (51-73)		
		59	61	n/a	n/a	n/a	0.175[j]	?	INCANT[h] (51-73)		
		49	42	MD	−3.92[c]	[−9.28; 1.44][c]	0.150[c, e]	▲	Slesnick 2013 (84-88)		
	Month 18	44	40	Hedges' g	−0.61[c]	[−1.05; −0.17][c]	**0.006**[c, e]	▲	Slesnick 2013 (84-88)		
	Month 24	41	38	Hedges' g	−0.63[c]	[−1.08; −0.18][c]	**0.006**[c, e]	▲	Slesnick 2013 (84-88)		
	Month 0 –24	n/a	n/a	n/a	n/a	n/a	≥ 0.05[i]	?	Slesnick 2013 (84-88)		

Table 11: Mental and behavioural disorders due to psychoactive substance use – results for all patient-relevant outcomes with assessable data (multipage Table)

No.	Outcome / Operationalisation / Length of follow-up	N ST	N COM	Effect measure	Effect estimate	[95 %-CI]	p-value	Direction of effect	Study	Conclusion on benefit at outcome level	Conclusion on benefit across outcomes
24	**Internalising problems**									⇔	
	**Internalising subscale of YSR**										
	Month 2	57	55	Hedges' g	−0.36[c]	[−0.73; 0.01][c]	0.058[c, e]	▲	Liddle 2018 (82, 83)		
	Month 0 – 2	57	56	n/a	n/a	n/a	**< 0.01**[d]	▲	Liddle 2018 (82, 83)		
	Month 3	49	45	Hedges' g	0.29[c]	[−0.12; 0.69][c]	0.164[c, e]	▼	Slesnick 2013 (84-88)		
	Month 4	55	54	MD	−1.07[c]	[−5.60; 3.46][c]	0.641[c, e]	▲	Liddle 2018 (82, 83)		
	Month 6	163[f]	183[f]	MD	−1.03[c]	[−2.78[c]; 0.72][c]	0.249[c, e]	▲	INCANT[g] (51-73)		
		42	42	MD	−2.49[c]	[−8.06; 3.08][c]	0.377[c, e]	▲	Slesnick 2013 (84-88)		
	Month 9	47	40	Hedges' g	0.07[c]	[−0.35; 0.49][c]	0.745[c, e]	▲	Slesnick 2013 (84-88)		
	Month 12	180[f]	202[f]	MD	−0.94[c]	[−0.77; 0.89][c]	0.312[c, e]	▲	INCANT[k] (51-73)		
		58	60	n/a	n/a	n/a	0.281[j]	?	INCANT[h] (51-73)		
		56	53	MD	−2.73[c]	[−6.45; 0.99][c]	0.148[c, e]	▲	Liddle 2018 (82, 83)		
		45	39	MD	0.61[c]	[−4.18; 5.40][c]	0.801[c, e]	▼	Slesnick 2013 (84-88)		
	Month 18	57	55	MD	−1.08[c]	[−4.71; 2.55][c]	0.556[c, e]	▲	Liddle 2018 (82, 83)		
		45	41	MD	1.30[c]	[−2.89; 5.49][c]	0.539[c, e]	▼	Slesnick 2013 (84-88)		
	Month 2 – 18	57	56	n/a	n/a	n/a	≥ 0.05[d]	?	Liddle 2018 (82, 83)		
	Month 24	43	41	Hedges' g	−0.06[c]	[−0.49; 0.37][c]	0.787[c, e]	▲	Slesnick 2013 (84-88)		
	Month 0 – 24	n/a	n/a	n/a	n/a	n/a	≥ 0.05[i]	?	Slesnick 2013 (84-88)		

Table 11: Mental and behavioural disorders due to psychoactive substance use – results for all patient-relevant outcomes with assessable data (multipage Table)

No.	Outcome<br>Operationalisation<br>Length of follow-up	N ST	N COM	Effect measure	Effect estimate	[95 %-CI]	p-value	Direction of effect	Study	Conclu-sion on benefit at outcome level	Conclu-sion on benefit across outcomes
	**Internalising subscale, parent / primary caretaker version of YSR, CBCL**										
	Month 3	49	47	Hedges' g	−0.15[c]	[−0.55; 0.25][c]	0.460[c]	▲	Slesnick 2013 (84-88)		
	Month 4	n/a	n/a	n/a	n/a	n/a	> 0.05	?	Waldron 2001 (89, 90)		
	Month 6	161[f]	180[f]	MD	−1.31[c]	[−3.52; 0.90][c]	0.245[c]	▲	INCANT[g] (51-73)		
		42	43	MD	−3.05[c]	[−7.43; 1.33][c]	0.169[c]	▲	Slesnick 2013 (84-88)		
	Month 7	n/a	n/a	n/a	n/a	n/a	> 0.05	?	Waldron 2001 (89, 90)		
	Month 9	47	41	Hedges' g;	−0.02[c]	[−0.44; 0.40][c]	0.933[c]	▲	Slesnick 2013 (84-88)		
	Month 12	49	42	MD	−2.17[c]	[−5.90; 1.56][c]	0.251[c]	▲	Slesnick 2013 (84-88)		
		171[f]	192[f]	MD	−0.88[c]	[−2.84; 1.08][c]	0.377[c]	▲	INCANT[k] (51-73)		
		58	61	n/a	n/a	n/a	0.096[j]	?	INCANT[h] (51-73)		
	Month 18	44	40	Hedges' g	−0.62[c]	[−1.06; −0.18][c]	0.005[c, e]	▲	Slesnick 2013 (84-88)		
	Month 24	41	38	Hedges' g	−0.53[c]	[−0.98; −0.08][c]	0.020[c, e]	▲	Slesnick 2013 (84-88)		
	Month 0 – 24	n/a	n/a	n/a	n/a	n/a	≥ 0.05[i]	?	Slesnick 2013 (84-88)		
**25**	**Symptoms of cannabis use disorder**									↗	
	**ADI-Light for Cannabis, number of symptoms**										
	Month 12	190	211	MD	−0.60[c]	[−0.99; −0.21][c]	**0.003**[c, e]	▲	INCANT (51-73)		
	Month 0 – 12	212	238	Cohen's d	1.27[d]	[0.51; 2.03]	**< 0.001**[d]	▲	INCANT (51-73)		
**26**	**Substance use (any substance, self-report)**									⇔	
	**GAIN, days without consumption of cannabis, alcohol and other substances**										
	Month 12	94	94[l]	MD	6[c]	n/a	> 0.05	▲	CYT (42-48)		
	Month 12	94	94[m]	MD	-8[c]	n/a	> 0.05	▼	CYT (42-48)		

Table 11: Mental and behavioural disorders due to psychoactive substance use – results for all patient-relevant outcomes with assessable data (multipage Table)

No.	Outcome / Operationalisation / Length of follow-up	N ST	N COM	Effect measure	Effect estimate	[95 %-CI]	p-value	Direction of effect	Study	Conclusion on benefit at outcome level	Conclusion on benefit across outcomes
**27**	**Substance use detected by laboratory tests**									⇔	
	**Urine analyses of substance use, THC**										
	Month 12	52	51	OR	0.97[c]	[0.40; 2.37][c]	> 0.05[c]	▲	INCANT (51-73)		
	**Urine analyses of substance use, all substances**										
	Month 4	n/a	n/a	n/a	n/a	n/a	> 0.05	?	Waldron 2001 (89, 90)		
	Month 7	n/a	n/a	n/a	n/a	n/a	> 0.05	?	Waldron 2001 (89, 90)		
**28**	**Substance use problem severity**									⇔	
	**PEI/PIC, 29 Items, subscore focusing on the psychological and behavioural depth of substance use involvement and related consequences in the previous 90 days**										
	Month 6	54	52	MD	1.04[c]	[−4.35; 6.43][c]	0.703[c, e]	▼	Dakof 2015 (49, 50)		
		59	60	MD	−5.80[c]	[−12.24; 0.64][c]	0.077[c, e]	▲	INCANT[h] (51-73)		
		29	27	Cohen's d	0.09[e]	n/a	> 0.05[e]	▲	INCANT[k] (51-73)		
	Month 9	59	60	MD	−5.40[c]	[−11.74; 0.94][c]	0.094[c, e]	▲	INCANT[h] (51-73)		
		29	27	Cohen's d	0.36[e]	n/a	> 0.05[e]	▲	INCANT[k] (51-73)		
	Month 12	46	45	MD	0.23[c]	[−6.23; 6.69][c]	0.944[c, e]	▼	Dakof 2015 (49, 50)		
		59	60	Cohen's d	n/a	n/a	0.082[j]	▲	INCANT[h] (51-73)		
	Month 12	29	27	Cohen's d	0.34[e]	n/a	> 0.05[e]	▲	INCANT[k] (51-73)		
	Month 18	47	46	MD	0.21[c]	[−6.29; 6.71][c]	0.949[c, e]	▼	Dakof 2015 (49, 50)		
	Month 24	51	43	Hedges' g	−0.22[c]	[−0.63; 0.19][c]	0.284[c, e]	▲	Dakof 2015 (49, 50)		
	Month 7 – 24	55	57	Cohen's d	0.54[d]	n/a	0.078[d]	▲	Dakof 2015 (49, 50)		

Table 11: Mental and behavioural disorders due to psychoactive substance use – results for all patient-relevant outcomes with assessable data (multipage Table)

No.	Outcome / Operationalisation / Length of follow-up	N ST	N COM	Effect measure	Effect estimate	[95 %-CI]	p-value	Direction of effect	Study	Conclusion on benefit at outcome level	Conclusion on benefit across outcomes
	**PEI/PIC, 29 Items, subscore focusing on the psychological and behavioural depth of substance use involvement and related consequences in the previous 30 days**										
	Month 0 – 18	112	112	n/a	n/a	n/a	**< 0.05**[d]	▲	Liddle 2018 (82, 83)		
	Month 2	57	55	Hedges' g	0.47	[0.10; 0.85][c]	**0.013**[c, e]	▼	Liddle 2018 (82, 83)		
	Month 0 –2	57	56	n/a	n/a	n/a	≥ 0.05[d]	?	Liddle 2018 (82, 83)		
	Month 4	55	54	MD	1.47[c]	[−3.65; 6.59][c]	0.571[c, e]	▼	Liddle 2018 (82, 83)		
	Month 12	56	53	MD	−2.62[c]	[−8.77; 3.53][c]	0.400[c, e]	▲	Liddle 2018 (82, 83)		
	Month 18	57	55	MD	−2.22[c]	[−7.34; 2.90][c]	0.392[c, e]	▲	Liddle 2018 (82, 83)		
	Month 2 –18	57	56	Cohen's d	0.51[d]	[> 0.12; < 0.90][c, n]	**< 0.01**[d]	▲	Liddle 2018 (82, 83)		
**29**	**Use of cannabis**									⇑	
	**TLFB, daily cannabis consumption in the previous 90 days**										
	Month 3	184[c]	207[c]	MD	−5.80[c]	[−12.04; 0.44][c]	0.068[c, e]	▲	INCANT (51-73)		
	Month 6	172[c]	193[c]	MD	−7.90[c]	[−14.45; −1.35][c]	**0.018**[c, e]	▲	INCANT (51-73)		
	Month 9	165[c]	186[c]	MD	−5.80[c]	[−12.68; 1.08][c]	0.098[c, e]	▲	INCANT (51-73)		
	Month 12	190	211	Cohen's d	−8.30[c]	[−14.83; −1.77][c]	**0.013**[e]	▲	INCANT (51-73)		
	Month 0 –12	212	238	Cohen's d	0.25[d]	n/a	0.07[d]	▼	INCANT (51-73)		
	**TLFB, number of joints in the previous 90 days**										
	Month 12	52	51	MD	−4.80[c]	[−42.93; 33.33][c]	0.803[c, e]	▲	INCANT (51-73)		
	Month 0 –12	55	54	Cohen's d	0.13[d]	n/a	0.50[d]	▼	INCANT (51-73)		

Table 11: Mental and behavioural disorders due to psychoactive substance use – results for all patient-relevant outcomes with assessable data (multipage Table)

No.	Outcome / Operationalisation / Length of follow-up	N ST	N COM	Effect measure	Effect estimate	[95 %-CI]	p-value	Direction of effect	Study	Conclusion on benefit at outcome level	Conclusion on benefit across outcomes
	**TLFB, daily cannabis consumption in the previous 30 days**										
	Month 0 –12	112	112	n/a	n/a	n/a	≥ 0.05[d]	?	Liddle 2008 (74-81)		
	**FORM 90D/TLFB; days with cannabis consumption in the previous 90 days**										
	Month 4	n/a	n/a	n/a	n/a	n/a	**< 0.025**[o]	▲	Waldron 2001 (89, 90)		
	Month 7	n/a	n/a	n/a	n/a	n/a	< 0.10[o]	?	Waldron 2001 (89, 90)		
	**ADI-Light for Cannabis, Response**										
	Month 12	52	51	OR	0.89[c]	[0.41; 1.95][c]	0.78[c, e]	▼	INCANT (51-73)		
	**ADI-Light for Cannabis, Abstinence**										
	Month 12	52	51	OR	1.13	[0.40; 3.19]	0.64	▲	INCANT (51-73)		
	**FORM 90D/TLFB, self reported minimal cannabis consumption**										
	Month 4	30	31	OR	1.87[c]	[0.65; 5.39][c]	0.265[c, e]	▲	Waldron 2001 (89, 90)		
	Month 7	30	31	OR	3.01[c]	[0.90; 10.11][c]	0.074[c, e]	▲	Waldron 2001 (89, 90)		
**30**	**Use of substances for which criteria for a substance use disorder are not met**									⇑⇓	
	**TLFB, days with cannabis consumption in the previous 30 days**										
	Month 0 –12	112	112	Cohen's d	0.32[d]	[> 0.00; < 2.03][c]	**< 0.05**[d]	▲	Liddle 2008 (74-81)		
	**TLFB, days with alcohol consumption in the previous 30 days**										
	Month 0 –12	112	112	n/a	n/a	n/a	≥ 0.05[d]	?	Liddle 2008 (74-81)		
	**FORM 90, percentage of substance abuse (defined as percentage of the previous 90 days)**										
	Month 3	48	45	MD	−3.20[c]	[−16.72; 10.32][c]	0.639[c, e]	▲	Slesnick 2013 (84-88)		
	Month 6	41	42	MD	5.00[c]	[−6.66; 16.66][c]	0.398[c, e]	▼	Slesnick 2013 (84-88)		

Table 11: Mental and behavioural disorders due to psychoactive substance use – results for all patient-relevant outcomes with assessable data (multipage Table)

No.	Outcome<br>Operationalisation<br>Length of follow-up	N ST	N COM	Effect measure	Effect estimate	[95 %-CI]	p-value	Direction of effect	Study	Conclu-sion on benefit at outcome level	Conclu-sion on benefit across outcomes
	Month 9	46	41	MD	18.60[c]	[6.16; 31.04][c]	**0.004**[c, e]	▲	Slesnick 2013 (84-88)		
	Month 12	46	40	MD	6.40[c]	[−6.77; 19.57][c]	0.337[c, e]	▼	Slesnick 2013 (84-88)		
	Month 18	45	41	MD	11.40[c]	[−2.92; 25.72][c]	0.117[c, e]	▼	Slesnick 2013 (84-88)		
	Month 24	39	41	MD	7.50[c]	[-9.39; 24.39][c]	0.379[c, e]	▼	Slesnick 2013 (84-88)		
	Month 0 –24	57	61	n/a	n/a	n/a	> 0.05[p]	?	Slesnick 2013 (84-88)		
	**ADI-Light, use of amphetamine in the previous 12 months**										
	Month 12	59	61	OR	1.17	[0.53; 2.59]	0.694	▼	INCANT[h] (51-73)		
	**ADI-Light, use of ecstasy in the previous 12 months**										
	Month 12	59	61	OR	1.01	[0.46; 2.24]	0.973	▼	INCANT[h] (51-73)		
	**ADI-Light, use of cocaine / crack in the previous 12 months**										
	Month 12	59	61	OR	1.42	[0.56; 3.66]	0.460	▼	INCANT[h] (51-73)		
	**ADI-Light, use of hallucinogen in the previous 12 months**										
	Month 12	59	61	OR	10.72	[1.56; 73.24]	**0.016**	▼	INCANT[h] (51-73)		
	**ADI-Light, use of sedatives /tranquilicer in the previous 12 months**										
	Month 12	59	61	OR	1.41	[0.41; 4.82]	0.581	▼	INCANT[h] (51-73)		
	**ADI-Light, use of heroin /opiates in the previous 12 months**										
	Month 12	59	61	OR	0.33	[0.00; 2.10]	0.107	▲	INCANT[h] (51-73)		

Table 11: Mental and behavioural disorders due to psychoactive substance use – results for all patient-relevant outcomes with assessable data (multipage Table)

No.	Outcome<br>Operationalisation<br>Length of follow-up	N<br>ST	N<br>COM	Effect<br>measure	Effect<br>estimate	[95 %-CI]	p-value	Direction of effect	Study	Conclusion on benefit at outcome level	Conclusion on benefit across outcomes

a. Hint of a greater benefit of ST compared to CBT across all outcomes based on the statistically significant effects in favour of ST for cannabis use disorder, symptoms of cannabis use disorder, and use of cannabis.
b. Proportional Odds Model (categories: remission / abuse / dependence)
c. own calculation
d. Latent Growth Curve Model
e. t-Test
f. N estimated from overall follow-up completion rates
g. whole sample
h. German sample
i. Multilevel Model (Random Coefficient Model)
j. ANCOVA, adjusted for Baseline, LOCF
k. Swiss sample
l. comparator: MET/CBT5
m. comparator: ACRA
n. based on effect measure and p-value
o. Regression analysis, adjusted for baseline value
p. Mixed-Effects Model
↔: no proof or indication or hint of a greater benefit or harm, one study
↗: hint of a greater benefit of ST, one study
⇑: indication of a greater benefit (supported by meta-analysis)
⇔: no proof or indication or hint of a greater benefit or harm (homogeneous result between several studies)
⇑⇓: no proof or indication or hint of a greater benefit or harm (heterogeneous result between several studies)
▲: effect in favour of ST
▼: effect to the disadvantage of ST
?: Direction of effect is unclear.
significant in **bold** ($p < 0.05$)

Table 11: Mental and behavioural disorders due to psychoactive substance use – results for all patient-relevant outcomes with assessable data (multipage Table)

No.	Outcome<br>Operationalisation<br>Length of follow-up	N ST	N COM	Effect measure	Effect estimate	[95 %-CI]	p-value	Direction of effect	Study	Conclusion on benefit at outcome level	Conclusion on benefit across outcomes

ACRA: Adolescent Community Reinforcement Approach; ADI: Adolescent Diagnostic Interview; CBCL: Child Behaviour Checklist; CI: Confidence interval; COM: Comparator; DSM-IV: Diagnostic and Statistical Manual of Mental Disorders (4th edition); GAIN: Global Appraisal of Individual Needs; LOCF: Last Observation Carried Forward; MD: Mean difference; MET/CBT5: Motivational Enhancement Therapy plus Cognitive Behavioural Therapy (5 sessions); N: number of analysed patients; n/a: not available or not specified in the study; OR: Odds Ratio; PEI: Personal Experience Inventory; PIC: Personal Involvement with Chemicals scale of the PEI; ST: Systemic Therapy; THC: tetrahydrocannabinol; TLFB: Timeline Follow-Back; vs.: versus; YSR: Youth Self-Report

A6.8 Forest plots – Class of mental disorders V: Mental and behavioural disorders due to psychoactive substance use

Systemic therapy (ST) vs. Cognitive behavioural therapy (CBT)
Externalising problems, YSR, 4 or 6 months
Random effects model - Knapp and Hartung

	ST			CBT					
Study	n	mean	SD	n	mean	SD	weight	Hedges' g	95% CI
Dakof 2015	54	47.44	10.79	52	46.37	9.93	16.5	0.10	[-0.28, 0.48]
INCANT	163	17.08	8.61	183	17.12	9.21	53.6	-0.00	[-0.22, 0.21]
Liddle 2018	55	53.10	11.25	54	52.28	10.80	16.9	0.07	[-0.30, 0.45]
Slesnick 2013	42	18.99	11.83	42	21.17	11.33	13.0	-0.19	[-0.62, 0.24]
Total	314			331			100.0	0.00	[-0.15, 0.16]

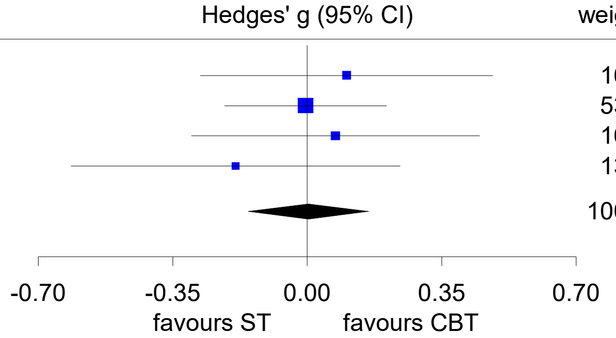

Heterogeneity: Q=1.15, df=3, p=0.764, I²=0%
Overall effect: Z-Score=0.06, p=0.959, Tau(Paule-Mandel)=0

Figure 2: Forest Plot for externalising problems (YSR) after 4 or 6 months; Systemic Therapy (ST) vs. Cognitive Behavioural Therapy (CBT); Mental and behavioural disorders due to psychoactive substance use

Systemic therapy (ST) vs. Cognitive behavioural therapy (CBT)
Externalising problems, CBCL, 6 months
Fixed effect model - inverse variance

	ST			CBT					
Study	n	mean	SD	n	mean	SD	weight	Hedges' g	95% CI
INCANT	161	18.44	10.68	180	18.76	12.39	80.4	-0.03	[-0.24, 0.19]
Slesnick 2013	42	21.58	14.67	43	27.69	13.55	19.6	-0.43	[-0.86, 0.00]
Total	203			223			100.0	-0.11	[-0.30, 0.08]

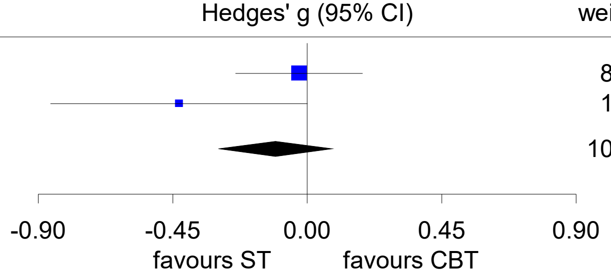

Heterogeneity: Q=2.69, df=1, p=0.101, I²=62.8%
Overall effect: Z-Score=-1.09, p=0.275

Figure 3: Forest Plot for externalising problems (CBCL) after 6 months; Systemic Therapy (ST) vs. Cognitive Behavioural Therapy (CBT); Mental and behavioural disorders due to psychoactive substance use

Systemic therapy (ST) vs. Cognitive behavioural therapy (CBT)
Externalising problems, YSR, 12 months
Random effects model - Knapp and Hartung (variance corr.)

	ST			CBT						
Study	n	mean	SD	n	mean	SD	Hedges' g (95% CI)	weight	Hedges' g	95% CI
Dakof 2015	46	45.63	8.79	45	46.16	9.04		13.7	-0.06	[-0.47, 0.35]
INCANT	180	15.38	9.07	202	15.86	8.80		57.3	-0.05	[-0.25, 0.15]
Liddle 2018	56	49.26	11.28	53	49.90	9.34		16.4	-0.06	[-0.44, 0.31]
Slesnick 2013	46	18.46	9.43	39	16.74	9.77		12.7	0.18	[-0.25, 0.61]
Total	328			339				100.0	-0.03	[-0.27, 0.22]

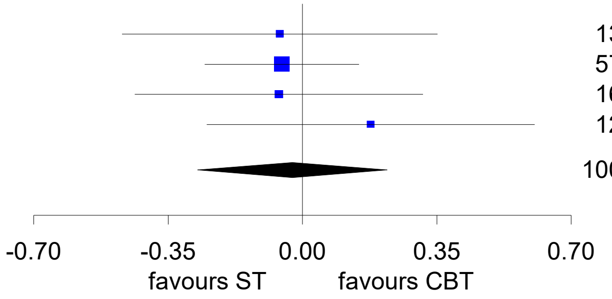

Heterogeneity: Q=1.00, df=3, p=0.800, I²=0%
Overall effect: Z-Score=-0.34, p=0.757, Tau(Paule-Mandel)=0

Figure 4: Forest Plot for externalising problems (YSR) after 12 months; Systemic Therapy (ST) vs. Cognitive Behavioural Therapy (CBT); Mental and behavioural disorders due to psychoactive substance use

Systemic therapy (ST) vs. Cognitive behavioural therapy (CBT)
Externalising problems, CBCL, 12 months
Fixed effect model - inverse variance

	ST			CBT					
Study	n	mean	SD	n	mean	SD	weight	Hedges' g	95% CI
INCANT	171	16.34	11.15	193	15.35	9.80	80.2	0.09	[-0.11, 0.30]
Slesnick 2013	49	20.60	12.85	42	24.52	12.80	19.8	-0.30	[-0.72, 0.11]
Total	220			235			100.0	0.02	[-0.17, 0.20]

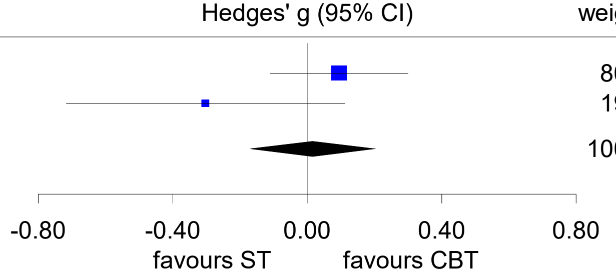

Heterogeneity: Q=2.83, df=1, p=0.092, I²=64.7%
Overall effect: Z-Score=0.17, p=0.867

Figure 5: Forest Plot for externalising problems (CBCL) after 12 months; Systemic Therapy (ST) vs. Cognitive Behavioural Therapy (CBT); Mental and behavioural disorders due to psychoactive substance use

Systemic therapy (ST) vs. Cognitive behavioural therapy (CBT)
Externalising problems, YSR, 18 months
Random effects model - Knapp and Hartung

	ST			CBT					
Study	n	mean	SD	n	mean	SD	weight	Hedges' g	95% CI
Dakof 2015	47	46.64	9.65	46	45.76	7.99	32.0	0.10	[-0.31, 0.51]
Liddle 2018	57	49.03	10.92	55	51.04	8.77	38.4	-0.20	[-0.57, 0.17]
Slesnick 2013	45	18.45	10.74	41	18.10	9.80	29.6	0.03	[-0.39, 0.46]
Total	149			142			100.0	-0.04	[-0.44, 0.37]

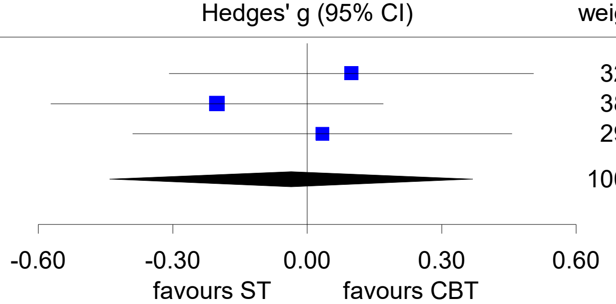

Heterogeneity: Q=1.28, df=2, p=0.526, I²=0%
Overall effect: Z-Score=-0.38, p=0.740, Tau(Paule-Mandel)=0

Figure 6: Forest Plot for externalising problems (YSR) after 18 months; Systemic Therapy (ST) vs. Cognitive Behavioural Therapy (CBT); Mental and behavioural disorders due to psychoactive substance use

Systemic therapy (ST) vs. Cognitive behavioural therapy (CBT)
Externalising problems, YSR, 24 months
Fixed effect model - inverse variance

	ST			CBT					
Study	n	mean	SD	n	mean	SD	weight	Hedges' g	95% CI
Dakof 2015	51	45.78	8.29	43	47.60	9.10	52.6	-0.21	[-0.62, 0.20]
Slesnick 2013	43	17.86	8.39	41	19.63	11.43	47.4	-0.18	[-0.60, 0.25]
Total	94			84			100.0	-0.19	[-0.49, 0.10]

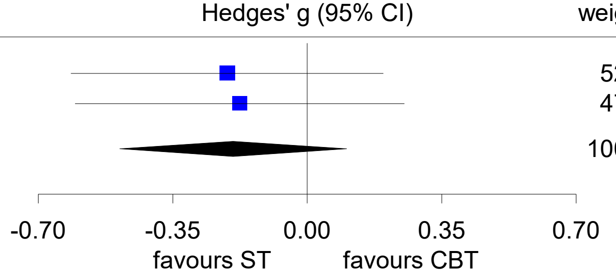

Heterogeneity: Q=0.01, df=1, p=0.914, I²=0%
Overall effect: Z-Score=-1.28, p=0.201

Figure 7: Forest Plot for externalising problems (YSR) after 24 months; Systemic Therapy (ST) vs. Cognitive Behavioural Therapy (CBT); Mental and behavioural disorders due to psychoactive substance use

Systemic therapy (ST) vs. Cognitive behavioural therapy (CBT)
Internalising problems, YSR, 4 or 6 months
Random effects model - Knapp and Hartung (variance corr.)

	ST			CBT					
Study	n	mean	SD	n	mean	SD	weight	Hedges' g	95% CI
INCANT	163	10.96	7.77	183	11.99	8.71	64.1	-0.12	[-0.34, 0.09]
Liddle 2018	55	48.07	10.98	54	49.14	12.84	20.3	-0.09	[-0.46, 0.29]
Slesnick 2013	42	18.32	11.63	42	20.81	13.95	15.6	-0.19	[-0.62, 0.24]
Total	260			279			100.0	-0.13	[-0.50, 0.24]

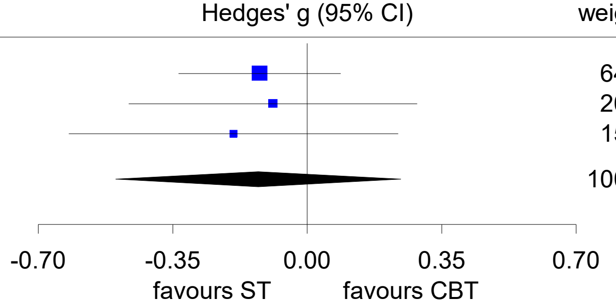

Heterogeneity: Q=0.13, df=2, p=0.938, I²=0%
Overall effect: Z-Score=-1.48, p=0.278, Tau(Paule-Mandel)=0

Figure 8: Forest Plot for internalising problems (YSR) after 4 or 6 months; Systemic Therapy (ST) vs. Cognitive Behavioural Therapy (CBT); Mental and behavioural disorders due to psychoactive substance use

Systemic therapy (ST) vs. Cognitive behavioural therapy (CBT)
Internalising problems, CBCL, 6 months
Fixed effect model - inverse variance

	ST			CBT					
Study	n	mean	SD	n	mean	SD	weight	Hedges' g	95% CI
INCANT	161	14.81	9.75	180	16.12	10.90	80.2	-0.13	[-0.34, 0.09]
Slesnick 2013	42	12.54	9.65	43	15.59	10.60	19.8	-0.30	[-0.73, 0.13]
Total	203			223			100.0	-0.16	[-0.35, 0.03]

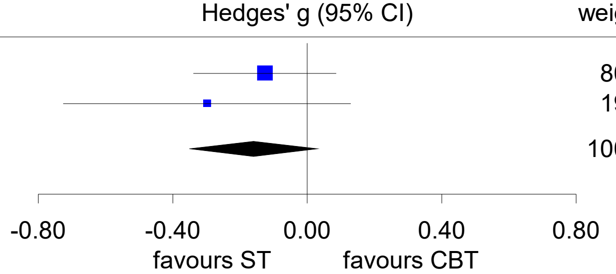

Heterogeneity: Q=0.50, df=1, p=0.480, I²=0%
Overall effect: Z-Score=-1.65, p=0.099

Figure 9: Forest Plot for internalising problems (CBCL) after 6 months; Systemic Therapy (ST) vs. Cognitive Behavioural Therapy (CBT); Mental and behavioural disorders due to psychoactive substance use

Systemic therapy (ST) vs. Cognitive behavioural therapy (CBT)
Internalising problems, YSR, 12 months
Random effects model - Knapp and Hartung

	ST			CBT					
Study	n	mean	SD	n	mean	SD	weight	Hedges' g	95% CI
INCANT	180	10.82	8.87	202	11.76	9.23	66.5	-0.10	[-0.30, 0.10]
Liddle 2018	56	45.72	8.87	53	48.45	10.67	18.9	-0.28	[-0.65, 0.10]
Slesnick 2013	45	16.58	11.20	39	15.97	10.80	14.6	0.05	[-0.37, 0.48]
Total	281			294			100.0	-0.11	[-0.41, 0.18]

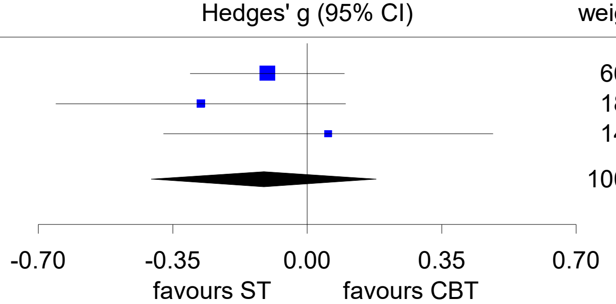

Heterogeneity: Q=1.32, df=2, p=0.516, I²=0%
Overall effect: Z-Score=-1.66, p=0.238, Tau(Paule-Mandel)=0

Figure 10: Forest Plot for internalising problems (YSR) after 12 months; Systemic Therapy (ST) vs. Cognitive Behavioural Therapy (CBT); Mental and behavioural disorders due to psychoactive substance use

Systemic therapy (ST) vs. Cognitive behavioural therapy (CBT)
Internalising problems, CBCL, 12 months
Fixed effect model - inverse variance

	ST			CBT					
Study	n	mean	SD	n	mean	SD	weight	Hedges' g	95% CI
INCANT	171	13.08	9.79	192	13.96	9.16	80.1	-0.09	[-0.30, 0.11]
Slesnick 2013	49	11.80	9.00	42	13.97	8.85	19.9	-0.24	[-0.65, 0.17]
Total	220			234			100.0	-0.12	[-0.31, 0.06]

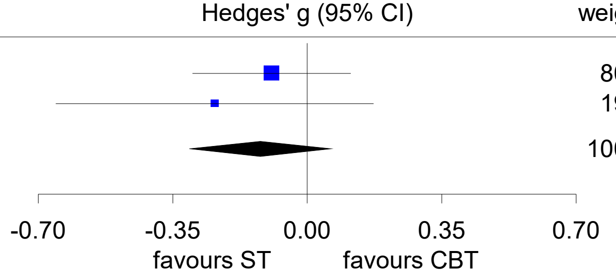

Heterogeneity: Q=0.39, df=1, p=0.530, I²=0%
Overall effect: Z-Score=-1.30, p=0.194

Figure 11: Forest Plot for internalising problems (CBCL) after 12 months; Systemic Therapy (ST) vs. Cognitive Behavioural Therapy (CBT); Mental and behavioural disorders due to psychoactive substance use

Systemic therapy (ST) vs. Cognitive behavioural therapy (CBT)
Internalising problems, YSR, 18 months
Fixed effect model - inverse variance

	ST			CBT					
Study	n	mean	SD	n	mean	SD	weight	Hedges' g	95% CI
Liddle 2018	57	45.96	9.79	55	47.04	9.57	56.6	-0.11	[-0.48, 0.26]
Slesnick 2013	45	15.93	10.43	41	14.63	8.98	43.4	0.13	[-0.29, 0.56]
Total	102			96			100.0	-0.01	[-0.28, 0.27]

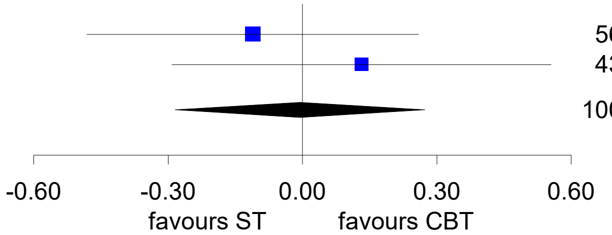

Heterogeneity: Q=0.71, df=1, p=0.398, I²=0%
Overall effect: Z-Score=-0.04, p=0.969

Figure 12: Forest Plot for internalising problems (YSR) after 18 months; Systemic Therapy (ST) vs. Cognitive Behavioural Therapy (CBT); Mental and behavioural disorders due to psychoactive substance use

Systemic therapy (ST) vs. Cognitive behavioural therapy (CBT)
Substance use problem severity, PEI/PIC, 4 or 6 months
Random effects model - Knapp and Hartung

	ST			CBT					
Study	n	mean	SD	n	mean	SD	weight	Hedges' g	95% CI
Dakof 2015	54	39.76	15.01	52	38.72	12.82	27.2	0.07	[-0.31, 0.45]
INCANT (Germany)	59	55.90	19.00	60	61.70	16.40	29.9	-0.32	[-0.69, 0.04]
INCANT (Switzerland)	29	55.00	15.01	27	56.60	14.83	15.0	-0.11	[-0.63, 0.42]
Liddle 2018	55	39.57	12.02	54	38.10	14.83	27.9	0.11	[-0.27, 0.48]
Total	197			193			100.0	-0.06	[-0.40, 0.28]

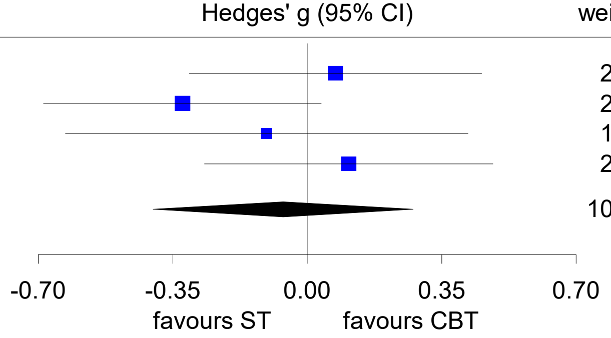

Heterogeneity: Q=3.33, df=3, p=0.343, I^2=10.0%
Overall effect: Z-Score=-0.59, p=0.598, Tau(Paule-Mandel)=0.063

Figure 13: Forest Plot for substance use problem severity after 4 or 6 months; Systemic Therapy (ST) vs. Cognitive Behavioural Therapy (CBT); Mental and behavioural disorders due to psychoactive substance use; missing standard deviation (SD) in INCANT (Switzerland) replaced by median of other studies

Systemic therapy (ST) vs. Cognitive behavioural therapy (CBT)
Substance use problem severity, PEI/PIC, 9 months
Fixed effect model - inverse variance

Study	ST n	ST mean	ST SD	CBT n	CBT mean	CBT SD	weight	Hedges' g	95% CI
INCANT (Germany)	59	52.00	17.90	60	57.40	17.00	68.0	-0.31	[-0.67, 0.05]
INCANT (Switzerland)	29	49.70	17.90	27	55.10	17.00	32.0	-0.30	[-0.83, 0.22]
Total	88			87			100.0	-0.31	[-0.60, -0.01]

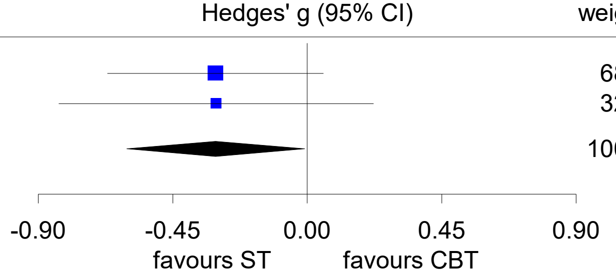

Heterogeneity: Q=0.00, df=1, p=0.993, I²=0%
Overall effect: Z-Score=-2.01, p=0.044

Figure 14: Forest Plot for substance use problem severity after 9 months; Systemic Therapy (ST) vs. Cognitive Behavioural Therapy (CBT); Mental and behavioural disorders due to psychoactive substance use; missing standard deviation (SD) in INCANT (Switzerland) replaced by median of other studies

Systemic therapy (ST) vs. Cognitive behavioural therapy (CBT)
Substance use problem severity, PEI/PIC, 12 months
Random effects model - Knapp and Hartung

	ST			CBT					
Study	n	mean	SD	n	mean	SD	weight	Hedges' g	95% CI
Dakof 2015	46	40.81	14.20	45	40.58	16.74	24.4	0.01	[-0.40, 0.43]
INCANT (Germany)	59	53.80	18.20	60	58.60	17.30	31.6	-0.27	[-0.63, 0.09]
INCANT (Switzerland)	29	51.50	14.74	27	57.00	17.30	14.8	-0.34	[-0.87, 0.19]
Liddle 2018	56	39.20	14.74	53	41.82	17.59	29.1	-0.16	[-0.54, 0.22]
Total	190			185			100.0	-0.18	[-0.41, 0.05]

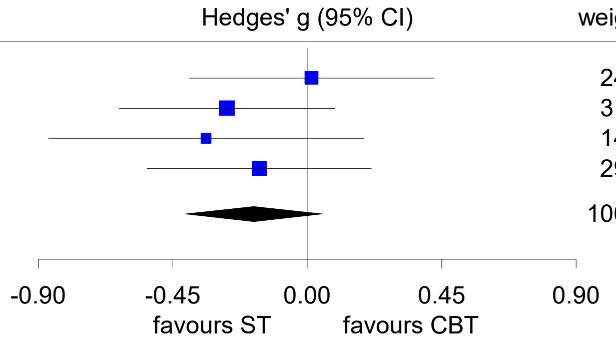

Heterogeneity: Q=1.45, df=3, p=0.694, I²=0%
Overall effect: Z-Score=-2.48, p=0.090, Tau(Paule-Mandel)=0

Figure 15: Forest Plot for substance use problem severity after 12 months; Systemic Therapy (ST) vs. Cognitive Behavioural Therapy (CBT); Mental and behavioural disorders due to psychoactive substance use; missing standard deviation (SD) in INCANT (Switzerland) replaced by median of other studies

Systemic therapy (ST) vs. Cognitive behavioural therapy (CBT)
Substance use problem severity, PEI/PIC, 18 months
Fixed effect model - inverse variance

	ST			CBT					
Study	n	mean	SD	n	mean	SD	weight	Hedges' g	95% CI
Dakof 2015	47	44.17	14.82	46	43.96	16.72	45.5	0.01	[-0.39, 0.42]
Liddle 2018	57	39.21	12.69	55	41.43	14.63	54.5	-0.16	[-0.53, 0.21]
Total	104			101			100.0	-0.08	[-0.36, 0.19]

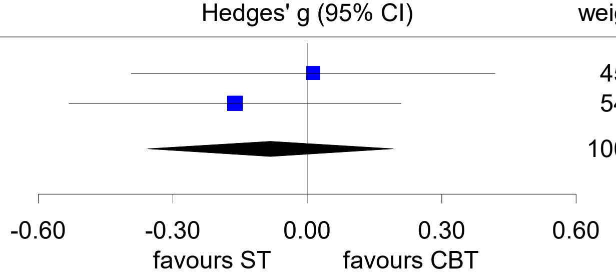

Heterogeneity: Q=0.39, df=1, p=0.535, I²=0%
Overall effect: Z-Score=-0.59, p=0.558

Figure 16: Forest Plot for substance use problem severity after 18 months; Systemic Therapy (ST) vs. Cognitive Behavioural Therapy (CBT); Mental and behavioural disorders due to psychoactive substance use

Systemic therapy (ST) vs. Cognitive behavioural therapy (CBT)
Use of cannabis, TLFB, 3 or 4 months
Fixed effect model - inverse variance (for presentation of the weights)

	ST			CBT				mean	
Study	n	mean	SD	n	mean	SD	weight	difference	95% CI
INCANT	184	39.40	32.50	207	45.20	30.20	88.2	-5.80	[-12.04, 0.44]
Waldron 2001	30	24.95	26.96	31	52.09	40.10	11.8	-27.14	[-44.24, -10.04]

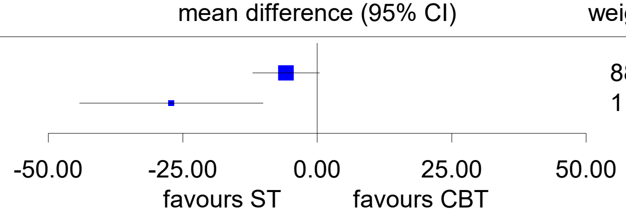

Heterogeneity: Q=5.28, df=1, p=0.022, I²=81.1%

Figure 17: Forest Plot for use of cannabis after 3 or 4 months; Systemic Therapy (ST) vs. Cognitive Behavioural Therapy (CBT); Mental and behavioural disorders due to psychoactive substance use; missing patient numbers in Waldron 2001 replaced with randomised patients

Systemic therapy (ST) vs. Cognitive behavioural therapy (CBT)
Use of cannabis, TLFB, 6 or 7 months
Fixed effect model - inverse variance

	ST			CBT					mean	
Study	n	mean	SD	n	mean	SD	mean difference (95% CI)	weight	difference	95% CI
INCANT	172	33.90	31.90	193	41.80	31.60		89.9	-7.90	[-14.43, -1.37]
Waldron 2001	30	40.10	40.07	31	51.13	37.35		10.1	-11.03	[-30.48, 8.42]
Total	202			224				100.0	-8.22	[-14.40, -2.03]

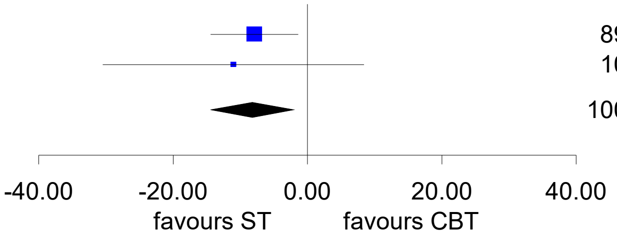

Heterogeneity: Q=0.09, df=1, p=0.765, I²=0%
Overall effect: Z-Score=-2.60, p=0.009

Figure 18: Forest Plot for use of cannabis after 6 or 7 months; Systemic Therapy (ST) vs. Cognitive Behavioural Therapy (CBT); Mental and behavioural disorders due to psychoactive substance use

References

1. Page MJ, McKenzie JE, Bossuyt PM, Boutron I, Hoffmann TC, Mulrow CD, et al. The PRISMA 2020 statement: an updated guideline for reporting systematic reviews. BMJ. 2021;372:n71.

2. Institut für Qualität und Wirtschaftlichkeit im Gesundheitswesen. Systemische Therapie als Psychotherapieverfahren bei Kindern und Jugendlichen; Abschlussbericht [Abschlussbericht/ -dokument]. 2023 [updated 27.01.2023; cited N21-03. Final report/document; 1507:[Available from: https://www.iqwig.de/download/n21-03_systemische-therapie-bei-kindern-und-jugendlichen_abschlussbericht_v1-0.pdf.

3. Wong SS, Wilczynski NL, Haynes RB. Comparison of top-performing search strategies for detecting clinically sound treatment studies and systematic reviews in MEDLINE and EMBASE. J Med Libr Assoc. 2006;94(4):451-5.

4. Lefebvre C, Glanville J, Briscoe S, Littlewood A, Marshall C, Metzendorf MI, et al. Cochrane Handbook for Systematic Reviews of Interventions; Version 6.2; Technical Supplement to Chapter 4: Searching for and selecting studies 2021 [Available from: https://training.cochrane.org/handbook/version-6.1/chapter-4-tech-suppl.

5. Eady AM, Wilczynski NL, Haynes RB, Team H. PsycINFO search strategies identified methodologically sound therapy studies and review articles for use by clinicians and researchers. J Clin Epidemiol. 2008;61(1):34-40.

6. Barbe RP, Bridge J, Birmaher B, Kolko D, Brent DA. Suicidality and its relationship to treatment outcome in depressed adolescents. Suicide Life Threat Behav. 2004;34(1):44-55.

7. Barbe RP, Bridge JA, Birmaher B, Kolko DJ, Brent DA. Lifetime history of sexual abuse, clinical presentation, and outcome in a clinical trial for adolescent depression. J Clin Psychiatry. 2004;65(1):77-83.

8. Birmaher B, Brent DA, Kolko D, Baugher M, Bridge J, Holder D, et al. Clinical outcome after short-term psychotherapy for adolescents with major depressive disorder. Arch Gen Psychiatry. 2000;57(1):29-36.

9. Brent DA, Baugher M, Birmaher B, Kolko DJ, Bridge J. Compliance with recommendations to remove firearms in families participating in a clinical trial for adolescent depression. J Am Acad Child Adolesc Psychiatry. 2000;39(10):1220-6.

10. Brent DA, Birmaher B, Kolko D, Baugher M, Bridge J. Subsyndromal depression in adolescents after a brief psychotherapy trial: course and outcome. J Affect Disord. 2001;63(1-3):51-8.

11. Brent DA, Holder D, Kolko D, Birmaher B, Baugher M, Roth C, et al. A clinical psychotherapy trial for adolescent depression comparing cognitive, family, and supportive therapy. Arch Gen Psychiatry. 1997;54(9):877-85.

12. Brent DA, Kolko DJ, Birmaher B, Baugher M, Bridge J. A clinical trial for adolescent depression: predictors of additional treatment in the acute and follow-up phases of the trial. J Am Acad Child Adolesc Psychiatry. 1999;38(3):263-70.

13. Brent DA, Kolko DJ, Birmaher B, Baugher M, Bridge J, Roth C, et al. Predictors of treatment efficacy in a clinical trial of three psychosocial treatments for adolescent depression. J Am Acad Child Adolesc Psychiatry. 1998;37(9):906-14.

14. Bridge JA, Barbe RP, Birmaher B, Kolko DJ, Brent DA. Emergent suicidality in a clinical psychotherapy trial for adolescent depression. Am J Psychiatry. 2005;162(11):2173-5.

15. Dietz LJ, Marshal MP, Burton CM, Bridge JA, Birmaher B, Kolko D, et al. Social problem solving among depressed adolescents is enhanced by structured psychotherapies. J Consult Clin Psychol. 2014;82(2):202-11.

16. Gaynor ST, Weersing VR, Kolko DJ, Birmaher B, Heo J, Brent DA. The prevalence and impact of large sudden improvements during adolescent therapy for depression: A comparison across cognitive-behavioral, family, and supportive therapy. J Consult Clin Psychol. 2003;71(2):386-93.

17. Kolko DJ, Brent DA, Baugher M, Bridge J, Birmaher B. Cognitive and family therapies for adolescent depression: treatment specificity, mediation, and moderation. J Consult Clin Psychol. 2000;68(4):603-14.

18. Renaud J, Brent DA, Baugher M, Birmaher B, Kolko DJ, Bridge J. Rapid response to psychosocial treatment for adolescent depression: a two-year follow-up. J Am Acad Child Adolesc Psychiatry. 1998;37(11):1184-90.

19. Stein D, Brent DA, Bridge J, Kolko D, Birmaher B, Baugher M. Predictors of parent-rated credibility in a clinical psychotherapy trial for adolescent depression. J Psychother Pract Res. 2001;10(1):1-7.

20. Lebowitz ER, Marin C, Martino A, Shimshoni Y, Silverman WK. Parent-based treatment as efficacious as cognitive-behavioral therapy for childhood anxiety: A randomized noninferiority study of supportive parenting for anxious childhood emotions. J Am Acad Child Adolesc Psychiatry. 2020;59(3):362-72.

21. Kircanski K, Peris TS. Exposure and response prevention process predicts treatment outcome in youth with OCD. J Abnorm Child Psychol. 2015;43(3):543-52.

22. Peris TS, Piacentini J. Optimizing treatment for complex cases of childhood obsessive compulsive disorder: a preliminary trial. J Clin Child Adolesc Psychol. 2013;42(1):1-8.

23. Peris TS, Rozenman MS, Sugar CA, McCracken JT, Piacentini J. Targeted Family Intervention for Complex Cases of Pediatric Obsessive-Compulsive Disorder: A Randomized Controlled Trial. J Am Acad Child Adolesc Psychiatry. 2017;56(12).

24. University of California. Family Focused Treatment of Pediatric Obsessive Compulsive Disorder (PFIT) 2021 [Available from: https://clinicaltrials.gov/ct2/show/NCT01409642.

25. Siqueland L, Rynn M, Diamond GS. Cognitive behavioral and attachment based family therapy for anxious adolescents: Phase I and II studies. J Anxiety Disord. 2005;19(4):361-81.

26. Gorrell S, Kinasz K, Hail L, Bruett L, Forsberg S, Lock J, et al. Rituals and preoccupations associated with bulimia nervosa in adolescents: Does motivation to change matter? Eur Eat Disord Rev. 2019;27(3):323-8.

27. Le Grange D, Lock J, Agras WS, Bryson SW, Jo B. Randomized Clinical Trial of Family-Based Treatment and Cognitive-Behavioral Therapy for Adolescent Bulimia Nervosa. J Am Acad Child Adolesc Psychiatry. 2015;54(11).

28. Matheson BE, Gorrell S, Bohon C, Agras WS, Le Grange D, Lock J. Investigating Early Response to Treatment in a Multi-Site Study for Adolescent Bulimia Nervosa. Frontiers in psychiatry. 2020;11:92.

29. Valenzuela F, Lock J, Le Grange D, Bohon C. Comorbid depressive symptoms and self-esteem improve after either cognitive-behavioural therapy or family-based treatment for adolescent bulimia nervosa. Eur Eat Disord Rev. 2018;26(3):253-8.

30. Stanford University. Study of Treatment for Adolescents With Bulimia Nervosa 2015 [Available from: https://ClinicalTrials.gov/show/NCT00879151.

31. Nyman-Carlsson E, Birgegard A, Engstrom I, Gustafsson SA, Nevonen L. Predictors of outcome among young adult patients with anorexia nervosa in a randomised controlled trial. Eur Eat Disord Rev. 2019;27(1):76-85.

32. Nyman-Carlsson E, Norring C, Engstrom I, Gustafsson SA, Lindberg K, Paulson-Karlsson G, et al. Individual cognitive behavioral therapy and combined family/individual therapy for young adults with Anorexia nervosa: A randomized controlled trial. Psychotherapy Research. 2020;30(8):1011-25.

33. Anorexia Bulimia Unit Child Adolescent Psychiatry Centre. The Gothenburg anorexia nervosa treatment study - a randomized control trial comparing individual cognitive behavioral therapy (I-CBT) and family therapy (FT) for young adults with anorexia nervosa 2019 [Available from: https://www.isrctn.com/ISRCTN25181390.

34. Perkins S, Schmidt U, Eisler I, Treasure J, Yi I, Winn S, et al. Why do adolescents with bulimia nervosa choose not to involve their parents in treatment? European Child and Adolescent Psychiatry, Supplement. 2005;14(7):376-85.

35. Schmidt U, Lee S, Beecham J, Perkins S, Treasure J, Yi I, et al. A randomized controlled trial of family therapy and cognitive behavior therapy guided self-care for adolescents with bulimia nervosa and related disorders. Am J Psychiatry. 2007;164(4):591-8.

36. Schmidt U, Lee S, Perkins S, Eisler I, Treasure J, Beecham J, et al. Do adolescents with eating disorder not otherwise specified or full-syndrome bulimia nervosa differ in clinical severity, comorbidity, risk factors, treatment outcome or cost? Int J Eat Disord. 2008;41(6):498-504.

37. The Health Foundation. A randomized controlled evaluation of the cost effectiveness of cognitive-behavioural guided self-care versus family therapy for adolescent bulimia nervosa in a catchment area-based population 2007 [Available from: https://www.isrctn.com/ISRCTN70120585.

38. Boyer BE, Doove LL, Geurts HM, Prins PJ, Van Mechelen I, Van der Oord S. Qualitative Treatment-Subgroup Interactions in a Randomized Clinical Trial of Treatments for Adolescents with ADHD: Exploring What Cognitive-Behavioral Treatment Works for Whom. PLoS One. 2016;11(3):e0150698.

39. Boyer BE, Geurts HM, Prins PJ, Van der Oord S. Two novel CBTs for adolescents with ADHD: the value of planning skills. Eur Child Adolesc Psychiatry. 2015;24(9):1075-90.

40. Boyer BE, Geurts HM, Prins PJ, Van der Oord S. One-year follow-up of two novel CBTs for adolescents with ADHD. Eur Child Adolesc Psychiatry. 2016;25(3):333-7.

41. University of Amsterdam. Training adolescents with ADHD to plan and organize: investigating short- and longterm effects of treatment. 2022 [Available from: https://trialsearch.who.int/Trial2.aspx?TrialID=NTR2142.

42. Burleson JA, Kaminer Y, Dennis ML. Absence of iatrogenic or contagion effects in adolescent group therapy: findings from the Cannabis Youth Treatment (CYT) study. Am J Addict. 2006;15 Suppl 1:4-15.

43. Dennis M, Godley SH, Diamond G, Tims FM, Babor T, Donaldson J, et al. The Cannabis Youth Treatment (CYT) Study: main findings from two randomized trials. J Subst Abuse Treat. 2004;27(3):197-213.

44. Dennis M, Titus JC, Diamond G, Donaldson J, Godley SH, Tims FM, et al. The Cannabis Youth Treatment (CYT) experiment: rationale, study design and analysis plans. Addiction. 2002;97 Suppl 1:16-34.

45. Diamond G, Godley SH, Liddle HA, Sampl S, Webb C, Tims FM, et al. Five outpatient treatment models for adolescent marijuana use: a description of the Cannabis Youth Treatment Interventions. Addiction. 2002;97 Suppl 1:70-83.

46. French MT, Roebuck MC, Dennis ML, Diamond G, Godley SH, Tims F, et al. The economic cost of outpatient marijuana treatment for adolescents: findings from a multi-site field experiment. Addiction. 2002;97 Suppl 1:84-97.

47. French MT, Roebuck MC, Dennis ML, Godley SH, Liddle HA, Tims FM. Outpatient marijuana treatment for adolescents. Economic evaluation of a multisite field experiment. Eval Rev. 2003;27(4):421-59.

48. Liddle HA. Multidimensional family therapy for adolescent cannabis users Rockville (USA): U.S. Department of Health and Human Services; 2002 [Available from: http://lib.adai.washington.edu/clearinghouse/downloads/Multidimensional-Family-Therapy-for-Adolescent-Cannabis-Users-207.pdf.

49. Dakof GA, Henderson CE, Rowe CL, Boustani M, Greenbaum PE, Wang W, et al. A randomized clinical trial of family therapy in juvenile drug court. J Fam Psychol. 2015;29(2):232-41.

50. University of Miami. Family-Based Juvenile Drug Court Services 2012 [Available from: https://ClinicalTrials.gov/show/NCT01668303.

51. Gantner A. [Multidimensional family therapy for adolescent clients with cannabis use disorders--Results and experience from the INCANT pilot study]. Prax Kinderpsychol Kinderpsychiatr. 2006;55(7):520-32.

52. Goorden M, van der Schee E, Hendriks VM, Hakkaart-van Roijen L. Cost-effectiveness of multidimensional family therapy compared to cognitive behavioral therapy for adolescents with a cannabis use disorder: Data from a randomized controlled trial. Drug Alcohol Depend. 2016;162:154-61.

53. Grichting E, Haug S, Nielsen P, Schaub M. INCANT Hauptstudie; Nationaler Schlussbericht Schweiz. Zürich: Institut für Sucht- und Gesundheitsforschung; 2011.

54. Har A, Bonnaire C. The end of INCANT: The transition phase from research to clinical work. Thérapie Familiale. 2013;34(4):529-41.

55. Hendriks V, van der Schee E, Blanken P. Treatment of adolescents with a cannabis use disorder: main findings of a randomized controlled trial comparing multidimensional family therapy and cognitive behavioral therapy in The Netherlands. Drug Alcohol Depend. 2011;119(1-2):64-71.

56. Hendriks V, van der Schee E, Blanken P. Matching adolescents with a cannabis use disorder to multidimensional family therapy or cognitive behavioral therapy: treatment effect moderators in a randomized controlled trial. Drug Alcohol Depend. 2012;125(1-2):119-26.

57. Hendriks VM, van der Schee E, Blanken P. [Multidimensional family therapy and cognitive behavioral therapy in adolescents with a cannabis use disorder: a randomised controlled study]. Tijdschrift voor Psychiatrie. 2013;55(10):747-59.

58. Lascaux M, Bastard N, Bonnaire C, Couteron J-P, Phan O. INCANT: Comparison of two formalized therapeutic models. Alcoologie et Addictologie. 2010;32(3):209-19.

59. Lascaux M, Phan O. [Comparison of European therapies for cannabis addiction among adolescents]. Encephale. 2015;41 Suppl 1:S21-8.

60. Phan O, Bonnaire C, Bastard N, Jouanne C. INCANT project. Psychotropes. 2009;14(3-4):137-56.

61. Phan O, Henderson CE, Angelidis T, Weil P, van Toorn M, Rigter R, et al. European youth care sites serve different populations of adolescents with cannabis use disorder. Baseline and referral data from the INCANT trial. BMC Psychiatry. 2011;11:110.

62. Phan O, Jouanne C, Monge S. A random clinical trial concerning the psychotherapy of adolescents addicted to cannabis. [French]. Ann Med Psychol (Paris). 2010;168(2):145-51.

63. Pol TM, Hendriks V, Rigter H, Cohn MD, Doreleijers TAH, Domburgh L, et al. Multidimensional family therapy in adolescents with a cannabis use disorder: Long-term effects on delinquency in a randomized controlled trial. Child Adolesc Psychiatry Ment Health. 2018;12(1):44.

64. Rigter H, Henderson CE, Pelc I, Tossmann P, Phan O, Hendriks V, et al. Multidimensional family therapy lowers the rate of cannabis dependence in adolescents: a randomised controlled trial in Western European outpatient settings. Drug Alcohol Depend. 2013;130(1-3):85-93.

65. Rigter H, Pelc I, Tossmann P, Phan O, Grichting E, Hendriks V, et al. INCANT: a transnational randomized trial of multidimensional family therapy versus treatment as usual for adolescents with cannabis use disorder. BMC Psychiatry. 2010;10:28.

66. Rowe C, Rigter H, Henderson C, Gantner A, Mos K, Nielsen P, et al. Implementation fidelity of Multidimensional Family Therapy in an international trial. J Subst Abuse Treat. 2013;44(4):391-9.

67. Schaub MP, Henderson CE, Pelc I, Tossmann P, Phan O, Hendriks V, et al. Multidimensional family therapy decreases the rate of externalising behavioural disorder symptoms in cannabis abusing adolescents: outcomes of the INCANT trial. BMC Psychiatry. 2014;14:26.

68. Tossmann P, Jonas B. Ergebnisbericht der kontrollierten Behandlungsstudie INCANT. Berlin: Delphi-Gesellschaft für Forschung, Beratung und Prokjektentwicklung; 2010.

69. Tossmann P, Jonas B, Rigter H, Gantner A. Treating adolescents with cannabis use disorder with Multidimensional Family Therapy (MDFT): Main results of a Randomized Controlled Trial (RCT). [German]. Sucht. 2012;58(3):157-66.

70. van der Pol TM, Henderson CE, Hendriks V, Schaub MP, Rigter H. Multidimensional Family Therapy Reduces Self-Reported Criminality Among Adolescents With a Cannabis Use Disorder. Int J Offender Ther Comp Criminol. 2018;62(6):1573-88.

71. Verbanck P, Pelc I, Glaesener M, Gladsteen Y, Spapen P. INCANT final report; Period: November 2006-April 2010. Brüssel: CHU Brugmann; 2010.

72. Erasmus Medical Centre. The effectiveness of outpatient multidimensional family therapy (MDFT) compared with outpatient treatment as usual in adolescents with a cannabis use disorder and other problem behaviour: a multicentre, trans-national randomised controlled trial 2018 [Yes. Available from: https://www.isrctn.com/ISRCTN51014277.

73. The Netherlands Ministry of Health, Welfare and Sport. The effectiveness of multidimensional family therapy (MDFT) versus cognitive behavioural therapy (CBT) in Dutch adolescents with a cannabis use disorder: a randomised controlled trial 2020 [Available from: https://www.isrctn.com/ISRCTN00179361.

74. Chinchilla P. Comorbidity as a moderator of process-outcome relations in individual and family therapy for adolescent substance abuse. New York: Fordham University; 2007. 6954 p.

75. Dauber S. Treatment focus in individual and family therapy for adolescent drug abuse. New York: Fordham University; 2004.

76. Henderson CE, Dakof GA, Greenbaum PE, Liddle HA. Effectiveness of multidimensional family therapy with higher severity substance-abusing adolescents: report from two randomized controlled trials. J Consult Clin Psychol. 2010;78(6):885-97.

77. Hogue A, Dauber S, Liddle HA, Samuolis J. Linking Session Focus to Treatment Outcome in Evidence-Based Treatments for Adolescent Substance Abuse. Psychotherapy: Theory, Research, Practice, Training. 2004;41(2):83-96.

78. Hogue A, Dauber S, Stambaugh LF, Cecero JJ, Liddle HA. Early therapeutic alliance and treatment outcome in individual and family therapy for adolescent behavior problems. J Consult Clin Psychol. 2006;74(1):121-9.

79. Hogue A, Henderson CE, Dauber S, Barajas PC, Fried A, Liddle HA. Treatment adherence, competence, and outcome in individual and family therapy for adolescent behavior problems. J Consult Clin Psychol. 2008;76(4):544-55.

80. Liddle HA, Dakof GA, Turner RM, Henderson CE, Greenbaum PE. Treating adolescent drug abuse: a randomized trial comparing multidimensional family therapy and cognitive behavior therapy. Addiction. 2008;103(10):1660-70.

81. Rowe CL, Liddle HA, Greenbaum PE, Henderson CE. Impact of psychiatric comorbidity on treatment of adolescent drug abusers. J Subst Abuse Treat. 2004;26(2):129-40.

82. Liddle HA, Dakof GA, Rowe CL, Henderson C, Greenbaum P, Wang W, et al. Multidimensional Family Therapy as a community-based alternative to residential treatment for adolescents with substance use and co-occurring mental health disorders. J Subst Abuse Treat. 2018;90:47-56.

83. University of Miami. Family-based and Adolescent Residential Drug Treatment 2012 [Available from: https://ClinicalTrials.gov/show/NCT01737632.

84. Guo X, Slesnick N, Feng X. Reductions in depressive symptoms among substance-abusing runaway adolescents and their primary caretakers: a randomized clinical trial. J Fam Psychol. 2014;28(1):98-105.

85. Guo X, Slesnick N, Feng X. Changes in Family Relationships among Substance Abusing Runaway Adolescents: A Comparison between Family and Individual Therapies. J Marital Fam Ther. 2016;42(2):299-312.

86. Slesnick N, Erdem G, Bartle-Haring S, Brigham GS. Intervention with substance-abusing runaway adolescents and their families: results of a randomized clinical trial. J Consult Clin Psychol. 2013;81(4):600-14.

87. Slesnick N, Guo X, Brakenhoff B, Feng X. Two-year predictors of runaway and homeless episodes following shelter services among substance abusing adolescents. J Adolesc. 2013;36(5):787-95.

88. Slesnick N, Guo X, Feng X. Change in parent- and child-reported internalizing and externalizing behaviors among substance abusing runaways: the effects of family and individual treatments. J Youth Adolesc. 2013;42(7):980-93.

89. French MT, Zavala SK, McCollister KE, Waldron HB, Turner CW, Ozechowski TJ. Cost-effectiveness analysis of four interventions for adolescents with a substance use disorder. J Subst Abuse Treat. 2008;34(3):272-81.

90. Waldron HB, Slesnick N, Brody JL, Turner CW, Peterson TR. Treatment outcomes for adolescent substance abuse at 4- and 7-month assessments. J Consult Clin Psychol. 2001;69(5):802-13.

91. Martín Andrés AM, Silva Mato AM. Choosing the optimal unconditioned test for comparing two independent proportions. Computational Statistics & Data Analysis. 1994;17(5):555-74.
